# Anemia and red blood cell transfusion in neurocritical care

**DOI:** 10.1186/cc7916

**Published:** 2009-06-11

**Authors:** Andreas H Kramer, David A Zygun

**Affiliations:** 1 Departments of Critical Care Medicine & Clinical Neurosciences, University of Calgary, Foothills Medical Center, 1403 29thSt. N.W., Calgary, AB, Canada, T2N 2T9; 2 Departments of Critical Care Medicine, Clinical Neurosciences, & Community Health Sciences, University of Calgary, Foothills Medical Center, 1403 29thSt. N.W., Calgary, AB, Canada, T2N 2T9

## Abstract

**Introduction:**

Anemia is one of the most common medical complications to be encountered in critically ill patients. Based on the results of clinical trials, transfusion practices across the world have generally become more restrictive. However, because reduced oxygen delivery contributes to 'secondary' cerebral injury, anemia may not be as well tolerated among neurocritical care patients.

**Methods:**

The first portion of this paper is a narrative review of the physiologic implications of anemia, hemodilution, and transfusion in the setting of brain-injury and stroke. The second portion is a systematic review to identify studies assessing the association between anemia or the use of red blood cell transfusions and relevant clinical outcomes in various neurocritical care populations.

**Results:**

There have been no randomized controlled trials that have adequately assessed optimal transfusion thresholds specifically among brain-injured patients. The importance of ischemia and the implications of anemia are not necessarily the same for all neurocritical care conditions. Nevertheless, there exists an extensive body of experimental work, as well as human observational and physiologic studies, which have advanced knowledge in this area and provide some guidance to clinicians. Lower hemoglobin concentrations are consistently associated with worse physiologic parameters and clinical outcomes; however, this relationship may not be altered by more aggressive use of red blood cell transfusions.

**Conclusions:**

Although hemoglobin concentrations as low as 7 g/dl are well tolerated in most critical care patients, such a severe degree of anemia could be harmful in brain-injured patients. Randomized controlled trials of different transfusion thresholds, specifically in neurocritical care settings, are required. The impact of the duration of blood storage on the neurologic implications of transfusion also requires further investigation.

## Introduction

A key paradigm in the management of neurocritical care patients is the avoidance of 'secondary' cerebral insults [1-3]. The acutely injured brain is vulnerable to systemic derangements, such as hypotension, hypoxemia, or fever, which may further exacerbate neuronal damage [4-7]. Thus, critical care practitioners attempt to maintain a physiologic milieu that minimizes secondary injury, thereby maximizing the chance of a favorable functional and neurocognitive recovery.

Anemia is defined by the World Health Organization as a hemoglobin (Hb) concentration less than 12 g/dl in women and 13 g/dl in men [8]. It is one of the most common medical complications encountered in critically ill patients, including those with neurologic disorders. About two-thirds of patients have Hb concentrations less than 12 g/dl at the time of intensive care unit (ICU) admission, with a subsequent decrement of about 0.5 g/dl per day [9-12]. The etiology of ICU-acquired anemia is multifactorial. Systemic inflammation reduces red blood cell (RBC) development by blunting the production of erythropoietin and interfering with the ability of erythroblasts to incorporate iron [13-17]. RBC loss is accelerated by frequent phlebotomy, reduced RBC survival, and occasional hemorrhage. Large volumes of fluid used during resuscitation, with resultant hemodilution, may also contribute to early reductions in Hb levels [18-22].

Anemia can easily be corrected with the use of allogeneic RBC transfusions. The proportion of patients receiving blood during their ICU stay varies from 20 to 44%, and those who are transfused receive an average of as many as five units [10,11,23,24]. However, two multi-center, randomized controlled trials (RCTs) and two large observational studies have shown the liberal use of blood transfusions, with the goal of maintaining relatively arbitrary Hb concentrations (e.g. 10 g/dl), to not only be ineffective at improving outcomes, but also potentially harmful [10,11,25,26]. Still, because impaired oxygen (O_2_) delivery is thought to be an important factor in secondary brain injury, it remains uncertain whether these findings can be broadly applied to neurocritical care patients. Accordingly, it remains common practice for clinicians to set target Hb levels at a minimum of 9 to 10 g/dl in this setting [27-29].

## Materials and methods

To describe the physiologic and clinical implications of anemia and transfusion in neurocritical care patients, we used the OVID interface to search MEDLINE from its inception until March 9, 2009. We combined the following MESH headings: (anemia OR blood transfusion OR hemodilution OR hematocrit OR hemoglobins) AND (stroke OR craniocerebral trauma OR subarachnoid hemorrhage OR cerebral hemorrhage OR cerebrovascular circulation OR cardiac surgical procedures OR coronary artery bypass). This search yielded 2137 English language publications dealing primarily with adults (>18 years old). Each abstract was reviewed, and both human and animal studies assessing the impact of anemia, hemodilution, or the use of RBC transfusions on a physiologic or clinical outcome were chosen for more detailed review. Relevant review articles and case reports were also included, and the references of selected papers were screened for additional publications. Clinical studies involving specific groups of neurocritical care patients were selected for inclusion in evidentiary tables.

## Results and discussion

### Physiologic implications of anemia

#### Cerebral blood flow and oxygen delivery

**Figure 1:**
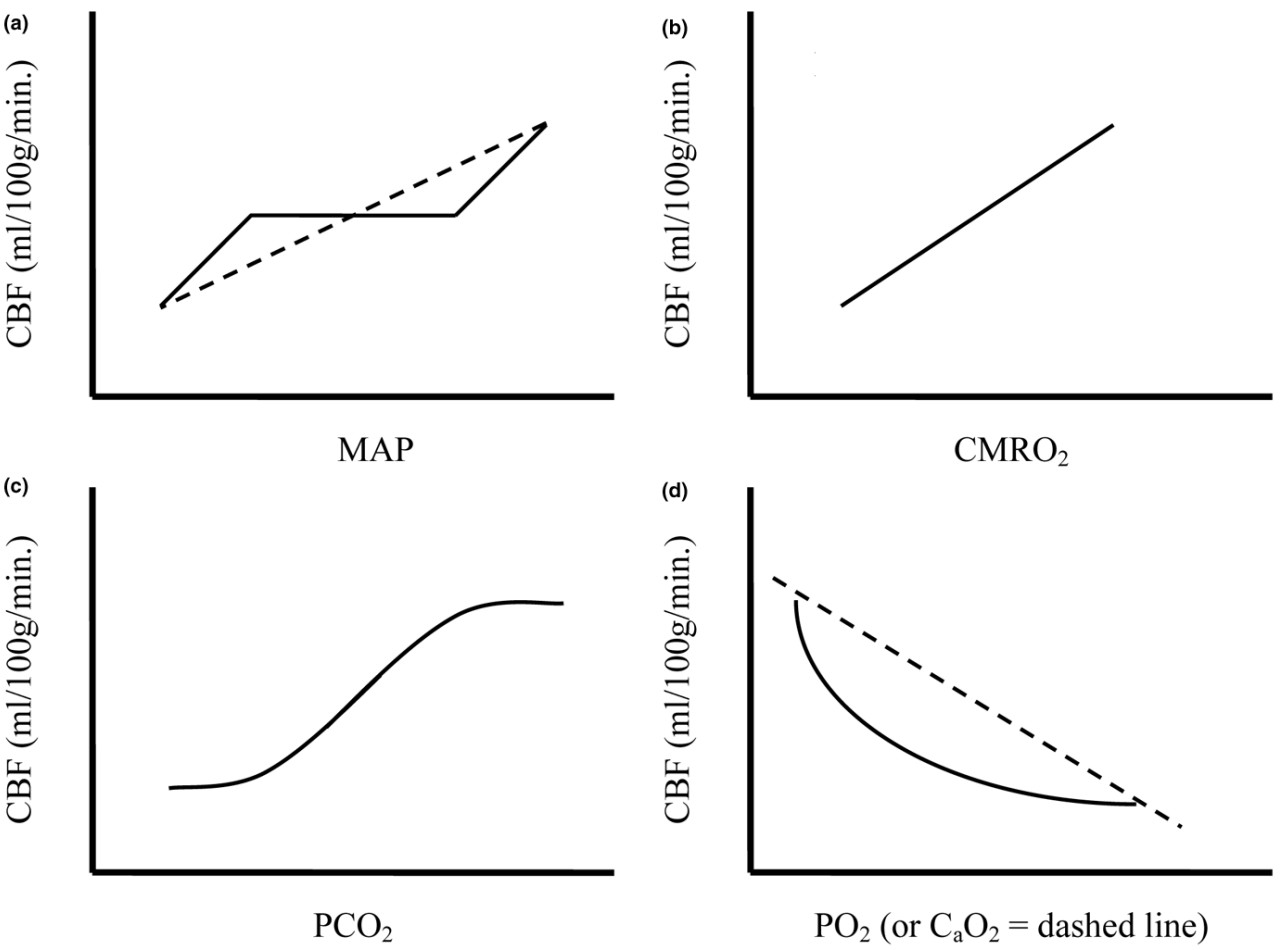
Physiologic parameters influencing cerebral blood flow **(a) **The effects of mean arterial blood pressure (MAP) (solid line = normal autoregulation; dashed line = deranged autoregulation), **(b) **cerebral metabolic rate (CMRO_2_), **(c) **partial pressure of carbon dioxide (PCO_2_), **(d) **partial pressure of oxygen (PO_2_) and arterial oxygen content (C_a_O_2_) (solid curved line = PO_2_; dashed line = C_a_O_2_) are shown. CBF = cerebral blood flow.

The amount of oxygen reaching specific organs is the product of local blood flow and the arterial oxygen content (C_a_O_2_). The latter is dependent on the Hb concentration and the degree to which it is saturated with O_2 _(S_a_O_2_), with a small amount of O_2 _also dissolved in blood. Thus, global systemic O_2 _delivery can be expressed by the following equation:



O_2 _delivery to the brain can be conceptualized using the same equation, but by substituting cerebral blood flow (CBF) for cardiac output (CO). Flow through the cerebral vasculature is determined by the cerebral perfusion pressure (CPP), the length and caliber of the vessels, and the viscosity of blood, as described by the Hagen-Poiseuille equation:



Regulation of CBF and cerebral O_2 _delivery in response to physiologic stressors is achieved largely by homeostatic variations in the caliber of cerebral vessels (the 'r' in the above equation; Figure 1).

CPP is the difference between mean arterial pressure and cerebral venous pressure; intracranial pressure is widely used as a surrogate for the latter. The response of the cerebral vasculature to changes in CPP is referred to as CBF autoregulation ('pressure-reactivity'). Cerebral arterioles vasoconstrict in response to raised CPP and vasodilate when there are reductions, thereby maintaining constant CBF (Figure 1a). Autoregulation is sometimes impaired in neurocritical care patients, such that CBF becomes directly dependent on CPP, making the brain more vulnerable to both hypo- and hyperperfusion [30-32].

There are numerous other stimuli that may modify cerebral vascular resistance and CBF. Both global and regional CBF are tightly coupled to metabolism. Thus, physiologic changes that lead to a reduction in cerebral metabolic rate (CMRO_2_) (e.g. hypothermia or sedation) will also proportionally reduce CBF (Figure 1b). In addition, CBF is influenced by variations in the partial pressures of carbon dioxide (PCO_2_; 'CO_2_-reactivity'), and to a lesser degree, O_2 _(PO_2_) (Figures 1c, d). CBF increases in response to a decrease in PO_2_, although this effect is probably minimal until the level approaches 60 mmHg [30].

In response to worsening anemia, neuronal O_2 _delivery is initially preserved both by the systemic cardiovascular response and mechanisms that are more specifically neuroprotective.

#### Cardiovascular response to anemia

A falling Hb concentration is sensed by aortic and carotid chemoreceptors, resulting in stimulation of the sympathetic nervous system, which in turn raises heart rate and contractility, thereby augmenting CO [33-35]. The reduction in blood viscosity results in a corresponding reduction in afterload, as well as enhanced flow through post-capillary venules, greater venous return, and increased preload [36-38]. Thus, stroke volume, CO, and blood pressure (as well as CPP) increase in response to isovolemic anemia. Tissues are further protected from falling O_2 _delivery because of their capacity to increase O_2 _extraction and maintain constant O_2 _consumption. In the brain, irreversible ischemia may not occur until the O_2 _extraction fraction (OEF) exceeds 75% [39-43]. Systemic anaerobic metabolism does not develop until the Hb concentration falls well below 5 g/dl in otherwise healthy individuals [44]. On the other hand, many neurocritical care patients have concomitant cardiac disease and left ventricular dysfunction which may prevent an appropriate increase in CO in response to sympathetic stimulation. This is commonly the case even in the absence of pre-existing heart disease; for example, among patients with acute 'high-grade' aneurysmal subarachnoid hemorrhage (SAH) (Hunt-Hess grades 3 to 5), more than one-third have regional left ventricular wall motion abnormalities detectable by echocardiography [45].

#### Cerebrovascular response to anemia

Apart from the increased flow produced by higher CPP and lower blood viscosity, anemia also induces cerebral vasodilatation [46-48]. When Hb (and therefore C_a_O_2_) falls, there appears to be a disproportionate increase in CBF in relation to other organs (Figure 1d) [49]. The mechanisms underlying this increase in vessel caliber are still being clarified, but include some of the same factors involved in CBF pressure-autoregulation; these have recently been reviewed in detail [46]. Importantly, anemia results in upregulation of nitric oxide (NO) production by perivascular neurons and vascular smooth muscle surrounding cerebral blood vessels. The importance of these pathways is supported by the observation that inhibition of NO synthase blunts hypoxia- and anemia-induced cerebral vasodilatation [50-52]. However, additional factors are undoubtedly involved [53-55]. Sympathetic β2 receptor stimulation is an example of one such mechanism that contributes to vasodilatation and maintenance of CBF [56]. Other biochemical mediators that are upregulated in the brain in response to anemia include vascular endothelial growth factor, hypoxia inducible factor 1α, and erythropoietin [46,57]. Although it seems likely that these mediators are neuroprotective, it remains possible that they could also have harmful pathophysiologic effects [46].

#### Compensatory mechanisms eventually fail

**Figure 2:**
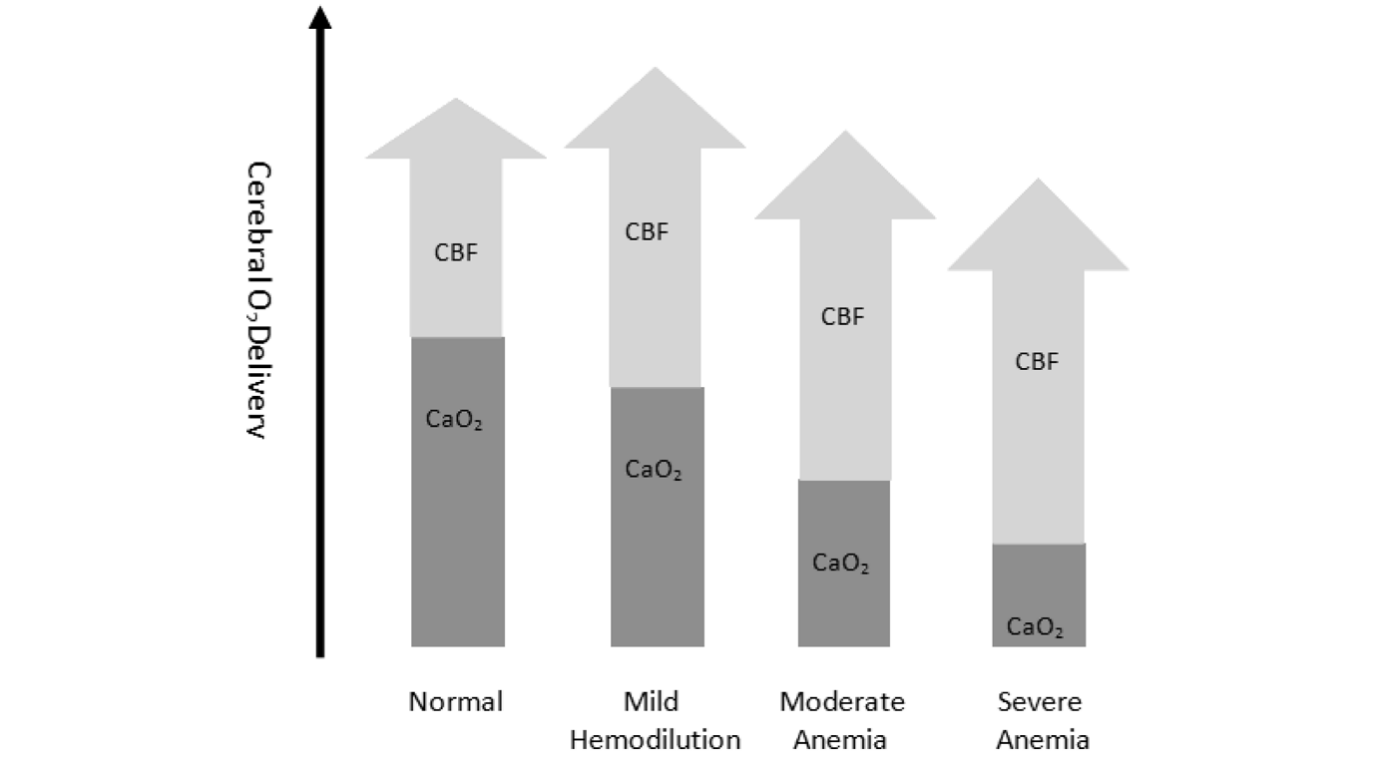
Effects of falling hemoglobin concentration on cerebral oxygen delivery. With mild hemodilution, it is theoretically possible that the resultant increase in cerebral blood flow (CBF) can raise overall O_2 _delivery. However, with further decrements in hemoglobin, the increment in CBF is insufficient to overcome the reduction in arterial oxygen content (C_a_O_2_).

As anemia worsens, the resultant increases in CBF and OEF eventually become insufficient to overcome the reduced C_a_O_2 _produced by a low Hb concentration (Figure 2). The point at which this threshold is reached is not clear and probably varies somewhat between patients. A sophisticated mathematical model based on animal data suggested that CMRO_2 _is well preserved in normal brain, even with severe reductions in Hb concentration. In contrast, penumbral brain appears to be much more vulnerable, with O_2 _delivery and CMRO_2 _progressively declining as Hb falls below 10 to 12 g/dl [58-62]. As with cerebral ischemia, impairment of the usual protective mechanisms induced by anemia has also been demonstrated as a result of brain trauma [63].

A study of euvolemic hemodilution in healthy human volunteers confirmed that even profound anemia (Hb about 5 g/dl) was relatively well tolerated; however, subtle abnormalities in neurocognitive testing began to emerge when Hb concentrations fell below 7 g/dl [64,65]. The co-existence of other physiologic stressors may also make anemia less tolerable; for example, experimental studies have found that cerebral O_2 _delivery is preserved in the presence of both severe anemia and hypotension individually, but not when they are both present [66,67]. Additionally, anemia-induced cerebral vasodilatation appears to interfere with the usual response to variations in PCO_2 _[47,68-70]. These observations raise concerns that relatively inadequate O_2 _delivery could occur at Hb levels well above 7 g/dl in critical care patients with cerebrovascular disease, pre-existing central nervous system pathology (e.g. an ischemic or 'traumatic' penumbra) or deranged regulation of CBF. Thus, there is strong physiologic rationale for believing that a restrictive transfusion threshold of 7 g/dl, although clearly safe in many critical care patients [25,26], may not be without risk in neurocritical care patients.

#### Risks of red blood cell transfusion

Even if anemia is harmful, this does not necessarily prove that liberal use of allogeneic RBCs to normalize Hb concentrations is justified. Emerging data indicates that stored blood has important differences from patients' own blood. A number of changes occur over time as RBCs are being stored; some of these alterations could have important implications after transfusion, and they are collectively referred to as the 'storage lesion'. Biochemical changes include reductions in ATP, loss of membrane phospholipids, and oxidative damage to proteins. The consequence is a gradual change in RBC appearance from the usual biconcave discs to irreversibly deformed and stellate-shaped spheroechinocytes [71,72]. Loss of RBC membrane function, as well as an increased tendency to adhere to endothelium, may interfere with microcirculatory flow [72,73]. RBC 2-3-diphosphoglycerate levels become depleted to the point of being essentially undetectable after one week of storage. Although levels are usually restored within 24 to 72 hours after transfusion, the transiently increased binding affinity of Hb interferes with the release of O_2 _for use by tissues [74].

Thus, although blood transfusions are generally given with the intention of raising O_2 _delivery, the storage-induced changes may prevent RBCs from achieving their intended purpose. For example, studies using gastric tonometry parameters as a surrogate for mesenteric perfusion have not shown improvements following transfusion [75,76]. Similarly, RBCs also appear to have little effect on skeletal muscle O_2 _tension in postoperative patients or on global O_2 _consumption in the critically ill [77,78].

Transfusion-related acute lung injury is now the most common cause of transfusion-related mortality reported to the Food and Drugs Administration [79]. Transfusion may have immunosuppressive effects, which are thought to be due to concomitant white blood cell transmission. Several studies have suggested a link between the use of allogeneic RBCs and both nosocomial infections and acute respiratory distress syndrome [80-83]. Alternatively, RCTs, where well-matched groups were transfused with differing intensities, have not yet convincingly confirmed these associations [25,26]. Furthermore, the risk of complications may be less since the implementation of universal leukoreduction in many jurisdictions [84].

It has been suggested that the use of fresher blood might further minimize the risks of transfusion, while also maximizing their physiologic effect. Results have been conflicting, and there is little data specifically in neurocritical care patients [71,75,76]. A recent animal study found fresh blood to be more effective at raising brain tissue oxygen tension (P_bt_O_2_) and preserving CBF in comparison to stored blood [85]. Alternatively, Weiskopf and colleagues performed isovolemic hemodilution to Hb concentrations of 55 to 74 g/L in healthy volunteers and then transfused them with autologous blood stored for either less than five hours or more than 14 days; neurocognitive test performance did not differ between the two groups [86].

### Anemia and RBC transfusion in specific neurocritical care settings

The importance of ischemia in causing secondary brain injury appears to vary for different neurocritical care conditions. For example, cerebral vasospasm and delayed infarction are major causes of neurologic deterioration in the two weeks following a ruptured cerebral aneurysm [87,88]. In contrast, the frequency and relevance of cerebral ischemia in the pathophysiology of traumatic brain injury (TBI) or intracerebral hemorrhage (ICH) continue to be debated [40,89-91]. Accordingly, the significance of anemia and optimal transfusion thresholds may not be consistent from one condition to the next.

#### Lessons from cardiac surgery

A great deal of what is known about the neurologic effects of anemia has been reported in the cardiac surgical literature. A substantial proportion of patients undergoing cardiac surgery receive blood transfusions, even though large volume hemorrhage is comparatively less common [92]. Perioperative stroke occurs in 1 to 6% of patients and is strongly associated with greater morbidity and mortality [93,94]. An even larger proportion (≥50%) develops at least transient neurocognitive dysfunction that is likely to be, at least in part, due to cerebral ischemia [95,96]. Thus, the prevention and treatment of cerebral ischemia is of major concern in the perioperative period.

We identified 12 studies assessing the association between perioperative Hb concentrations and subsequent neurologic complications (Table 1). When defined as an Hb concentration less than 12.5 g/dl, about one-quarter of patients are anemic preoperatively [97]. Blood loss and hemodilution during cardiopulmonary bypass usually lead to nadir intraoperative Hb concentrations of 7.0 to 8.5 g/dl; levels at ICU admission are typically 8.5 to 9.5 g/dl [98]. Several, but not all, studies have suggested that the degree of Hb reduction is an independent predictor of stroke, delirium, neurocognitive dysfunction, and other adverse outcomes [97-108] (Table 1). Although it has not been proven with certainty that these relations are causative, it seems prudent to avoid major reductions in Hb as best as possible with relevant blood-conservation strategies [109-113].

**Table 1:** Adult studies assessing the association between anemia and the development of perioperative stroke or cognitive dysfunction among patients undergoing cardiac surgery

**Study**	**Patients**	**Design and setting**	**Multivariable analysis**	**Exposure**	**Outcome**	**Main result**
Karkouti and colleagues [97]	10,179	Retrospective(prospective database) Single-center	Logistic regression	Maximum decrease intraoperative Hb compared with baseline	Composite of in-hospital death, stroke (new persistent postoperative neurologic deficit), or dialysis-dependent renal failure	>50% decrement in Hb independently associated with composite outcome

Bell and colleagues [98]	36,658 (CABG)	Retrospective (prospective database) Multi-center	Logistic regression	Preoperative Hb	Postoperative stroke (not further defined)	No significant association between Hb and stroke

Karkouti and colleagues [99]	3286 (CABG)	Retrospective Multi-center	Logistic regression and propensity scores	Preoperative anemia (Hb <12.5 g/dl)	Postoperative stroke (new neurologic deficit)	- Risk of stroke 1.1% in non-anemic pts vs. 2.8% in anemic patients - Trend towards more stroke among anemic patients in propensity-matched analysis

Chang and colleagues [100]	288	Retrospective Single-center	Logistic regression	Postoperative Hct <30%	Delirium (*DSM-IV *criteria)	Postoperative hct <30% associated with development of delirium (OR = 2.2, *P *= 0.02)

Kulier and colleagues [101]	4804	Retrospective (prospective database) Multi-center	Logistic regression	Preoperative Hb	'Cerebral outcomes' = stroke or encephalopathy (not further defined)	- Each 10 g/L Hb reduction associated with 15% increase in risk of non-cardiac (renal or CNS) complications - Association stronger for renal complications

Matthew and colleagues [102]	121 (CABG; age >65)	Prospective RCT Single-center	Logistic regression	Comparison of hemodilution to hct of ≥27% vs. 15 to 18%	Six-week postoperative neurocognitive function (battery of 5 tests)	- Trial stopped early because of unusually high rate of complications in both groups - Significant interaction between age and hct; more neurocognitive deficits among older patients with low hct

Cladellas and colleagues [103]	201 (VR)	Retrospective (prospective database) Single-center	None	Preoperative anemia (Hb <12 g/dl)	New permanent stroke or transient ischemic attack (not further defined)	- Risk of TIA or stroke 9.5% in anemic patients vs. 4.4% in non-anemic

Giltay and colleagues [104]	8139 (CABG)	Retrospective Single-center	Logistic regression	Lowest hematocrit first 24 hours ICU	Psychotic symptoms (hallucinations and/or delusions)	Hct <25% associated with psychosis (OR = 2.5 vs. hct >30%, CI 1.2 to 5.3)

Karkouti and colleagues [105]	10,949	Retrospective (prospective database) Single-center	Logistic regression	Nadir intraoperative hct	Postoperative stroke (new persistent postoperative neurologic deficit) that was present on emergence from anesthesia	Each 1% hct reduction associated with OR = 1.1 for stroke (*P *= 0.002)

Habib and colleagues [106]	5000	Retrospective (prospective database) Single-center	None	Nadir intraoperative hct	Transient or permanent postoperative stroke (not further defined)	Risk of TIA or stroke 5.4% in quintile with lowest hct vs. 1.3% in quintile with highest hct (*P *< 0.001)

DeFoe and colleagues [107]	6980 (CABG)	Retrospective (prospective database) Multi-center	Logistic regression	Nadir intraoperative hct	Intra- or postoperative stroke (new focal neurologic deficit which appears and is still at least partially evident more than 24 hours after onset; occurs during or following CABG)	No statistically significant association between hct and stroke

Van Wermeskerken and colleagues [108]	2804 (CABG)	Retrospective Single-center	Logistic regression	Nadir intraoperative hct	Adverse neurologic outcomes: stroke, coma, or TIA; verified retrospectively by neurologist	No significant association between hct and outcome

CABG = coronary artery bypass grafting; CI = confidence interval; CNS = central nervous system; Hb = hemoglobin; hct = hematocrit; ICU = intensive care unit; OR = odds ratio; RCT = randomized controlled trial; TIA = transient ischemic attack; VR = valve replacement

A recent RCT involving 121 elderly patients undergoing coronary artery bypass compared two intraoperative hematocrit targets (15 to 18% vs. ≥ 27%) [102]. The study was terminated early because of high complication rates in both groups; however, a greater degree of postoperative neurocognitive dysfunction was observed among patients managed with more extreme hemodilution. In addition, although not necessarily directly applicable to adults, further evidence that excessive hemodilution may have harmful neurologic effects comes from the neonatal literature. Combined data from two RCTs suggested that hematocrit levels below 23.5% during cardiopulmonary bypass were associated with impaired psychomotor development at one year of age [114-116].

Whether using RBC transfusions to maintain higher perioperative Hb levels helps avoid neurologic complications remains uncertain. For example, although Karkouti and colleagues found nadir hematocrit levels during cardiopulmonary bypass to be a predictor of stroke in a multivariable analysis, the same was also true for the perioperative use of transfusions [105]. An association between transfusion and focal or global neurologic deficits has been confirmed in numerous other studies (Table 2) [117-125].

**Table 2:** Adult studies assessing the association between transfusion and the development of perioperative stroke or cognitive dysfunction among patients undergoing cardiac surgery

**Study**	**Patients**	**Design and setting**	**Multivariable analysis**	**Exposure**	**Outcome**	**Main result**
Brevig and colleagues [117]	2531	Retrospective (prospective database) Single-center	None	Any blood product transfusion	Postoperative CVA (not further defined)	Despite reduction in proportion of patients transfused over time (43% in 2003 vs. 18% in 2007), no change in proportion of patients with CVA (0.8 to 1.5%)

Ngaage and colleagues [118]	383 (≥80 years old)	Retrospective (prospective database) Single-center	Logistic regression	Any blood product transfusion	Neurologic complications (confusion/agitation, seizures, TIA, RIND, stroke, or coma)	Transfusion associated with neurologic complications (OR = 3.6 vs. no transfusion, *P *= 0.003)

Murphy and colleagues [119]	8518	Retrospective Single-center	Logistic regression and propensity scores	Any perioperative RBC transfusion	Composite of MI, stroke (permanent or transient), or renal failure	RBC transfusion was associated with composite outcome (OR = 3.35 for transfusion vs. no transfusion; *P *< 0.0001)

Whitson and colleagues [120]	2691	Retrospective (prospective database) Single-center	Logistic regression	Any RBC transfusion	CVA (not further defined)	RBC transfusion was associated with CVA (OR = 1.7, *P *= 0.01)

Norkiene and colleagues [121]	1367	Retrospective Single-center	Logistic regression	Any RBC transfusion	Delirium (*DSM-IV *criteria)	Postoperative RBC transfusion was associated with delirium (OR = 4.6, *P *< 0.001)

Koch and colleagues [122]	11,963 (CABG)	Retrospective (prospective database) Single-center	Logistic regression	Total number of units of RBCs transfused	Focal or global neurologic deficits or death without awakening	RBC transfusion was associated with stroke (OR = 1.73 for each unit RBCs; *P *< 0.0001)

Stamou and colleagues [123]	49 JW patients	Retrospective Single-center	196 controls Logistic regression and propensity scores	Any RBC transfusion Nadir Hb not reported	Perioperative stroke	No statistically significant difference in risk of stroke between JWs refusing RBCs and transfused control patients

Karkouti and colleagues [105]	10,949	Retrospective (prospective database) Single-center	Logistic regression	Total number of units of blood product	New perioperative persistent postoperative neurological deficit	Transfusion was associated with stroke (OR = 1.02 for each unit RBCs; *P *= 0.01)

Bucerius and colleagues [124]	16,184	Retrospective (prospective database) Single-center	Logistic regression	Any perioperative RBC transfusion	Temporary or permanent focal or global neurologic deficit	'High transfusion requirement' ((≥1000 ml) was associated with stroke (OR = 6.04; *P *< 0.0001)

D'Ancona and colleagues [125]	9916 (CABG)	Retrospective (prospective database) Single-center	Logistic regression	Any blood product transfusion	New temporary or permanent, focal or global neurologic deficit	Transfusion was associated with stroke (OR = 1.59 vs. no transfusion; *P *= 0.002)

CABG = coronary artery bypass grafting; CVA = cerebrovascular accident; Hb = hemoglobin; JW = Jehovah's Witness; MI = myocardial infarction; OR = odds ratio; RBC = red blood cell; RIND = reversible ischemic neurologic deficit; TIA = transient ischemic attack.

One study compared clinical outcomes, including the risk of perioperative stroke, between 49 Jehovah's Witnesses who underwent cardiac surgery without blood products and a matched control group of 196 patients, in whom RBC transfusions were used. No significant differences were observed; however, only nine patients in total experienced a stroke, such that this study lacked statistical power to detect a difference. The severity of anemia in Jehovah's Witness patients was not reported [123].

In a large, single-center, retrospective study, Koch and colleagues explored whether the association between RBCs and worse outcomes could be related to the duration of blood storage. Outcomes were compared among cardiac surgical patients depending on whether they were transfused with exclusively 'newer' (≤14 days old; median 11 days) or 'older' (>14 days old; median 20 days) blood during the perioperative period [126]. In-hospital mortality and postoperative complications, including sepsis, renal failure, and need for mechanical ventilation, were greater among patients receiving older blood. However, there was no significant difference in the incidence of stroke and coma.

In summary, there remains uncertainty concerning optimum Hb levels for neuroprotection of patients undergoing cardiac surgery. Many intensivists routinely employ a postoperative transfusion threshold of 7 g/dl, although this may not be the optimum Hb level for the avoidance of neurologic complications. By necessity, the recommendations of published consensus guidelines are relatively non-specific, and state that it is "not unreasonable to transfuse red cells in certain patients with critical noncardiac end-organ ischemia whose Hb levels are as high as 10 g/dl" [111]. Funding was recently secured in the UK for a multi-center RCT comparing transfusion triggers of 7.5 vs. 9 g/dl [92].

#### Traumatic brain injury

The majority of patients dying from severe TBI have histologic evidence of ischemic damage [127]. Early global CBF reductions occur in many patients, often to levels that are considered to be in the ischemic range [128,129]. Reductions in both jugular venous O_2 _saturation (S_jv_O_2_) and P_bt_O_2 _are not only common, but their frequency and depth are predictive of worse outcomes [130-133]. However, the fall in CBF may be appropriate for a corresponding drop in metabolic rate [134,135]. Recent studies using positron emission tomography (PET) have suggested that although ischemia does occur, it is less common than previously thought. Furthermore, much of the 'metabolic distress' detected by multimodal monitoring (S_jv_O_2_, P_bt_O_2_, and microdialysis parameters) is not necessarily attributable to classical ischemia [39,134,135].

On the other hand, there appears to be a great deal of regional heterogeneity in CBF and CMRO_2 _[136]. Even if the overall ischemic brain volume is relatively small, certain vulnerable regions may still benefit from enhanced O_2 _delivery [137]. As with cardiac surgical patients, relatively extreme reductions in Hb are likely to be deleterious. A recent animal model found that although isovolemic hemodilution to Hb concentrations of 5 to 7 g/dl resulted in an overall increase in CBF, it produced larger contusion volumes, more apoptosis, and lower P_bt_O_2 _[138].

Potentially beneficial physiologic effects of transfusion have been shown in four studies of patients with severe TBI [139-142], each of which demonstrated that P_bt_O_2 _increases following the administration of RBCs (Table 3) [139]. However, this increment was inconsistent, relatively small and often of questionable clinical importance. Of concern, in some cases there was even a reduction in P_bt_O_2_. It is possible that some of the variation in the cerebral effects of transfusion could be, in part, attributable to the variable age of transfused blood. Leal-Noval and colleagues recently found that only those patients having received RBCs less than 14 days old had a statistically significant improvement in P_bt_O_2 _one hour after transfusion [141]. Although these results are intriguing, they are too premature to influence clinical practice and require confirmation in larger studies. Just because P_bt_O_2 _rises, does not necessarily mean that CMRO_2 _has increased. On the contrary, Zygun and colleagues found no improvement in cerebral lactate to pyruvate ratio (LPR – a marker of ischemia and 'metabolic distress') in response to transfusion, despite an increment in P_bt_O_2 _[142].

**Table 3:** Clinical studies assessing the impact of anemia or RBC transfusions on P_bt_O_2 _and other physiologic parameters in brain-injured patients

**Study**	**Patients**	**Design**	**Baseline**	**Intervention**	**Main findings**
Smith and colleagues [139]	23 TBI 12 SAH	Retrospective (prospective database)	Hb = 8.7 g/dl P_bt_O_2 _= 24.4 mmHg	Any RBC transfusion (number of units not specified *a priori*; 80% received ≥1 unit; mean Hb increased to 10.2 g/dl) General transfusion threshold Hb <10 g/dl or hct <30% (no protocol)	- Mean increment in P_bt_O_2 _3.2 mmHg (15%) - Increment not related to baseline P_bt_O_2_ - P_bt_O_2 _decreased in 9/35 patients (26%)

Leal-Noval and colleagues [140]	51 TBI	Prospective observational	Hb = 9.0 g/dl P_bt_O_2 _= 24.4 mmHg	1 or 2 units RBCs (number of units not specified *a priori*; 52% received 2 units; mean Hb increased to 10.6 g/dl) General transfusion threshold Hb <10 g/dl (no protocol)	- Mean increment in P_bt_O_2 _3.8 mmHg (16%) - Increment larger at lower baseline P_bt_O_2_ - P_bt_O_2 _decreased in 13/51 patients (25%)

Leal-Noval and colleagues [141]	66 TBI (males)	Prospective observational	Hb = 8.9 g/dl P_bt_O_2 _= 21.3 to 26.2 mmHg	1 or 2 units RBCs number of units not specified *a priori*; 59% received 2 units; mean Hb increased to 10.2 g/dl) General transfusion threshold Hb <9.5 g/dl (no protocol)	- Newer units of blood (≤14 days) resulted in greater mean increment in P_bt_O_2 _(3.3 mmHg (16%) vs. 2.1 mmHg (8%)) - P_bt_O_2 _decreased only in patients receiving older blood (>19 days)

Zygun and colleagues [142]	30 TBI	Prospective RCT	Hb = 8.2 g/dl P_bt_O_2 _= 18.8 mmHg	Randomized to transfusion thresholds of 8, 9, or 10 g/dl; 2 units RBCs administered over 2 hours (mean Hb increased to 10.1 g/dl)	- Mean increment in P_bt_O_2 _2.2 mmHg (12%) - Increment in P_bt_O_2 _most prominent when LPR >25 - P_bt_O_2 _decreased in 13/30 patients (43%) - No effect on S_jv_O_2 _or microdialysis parameters

Ekelund and colleagues [162]	8 SAH (TCD-vaso-spasm)	Prospective interventional	Hb = 11.9 g/dl	Isovolemic hemodilution (venesection with infusion of dextran 70 and 4% albumin) to mean Hb of 9.2 g/dl	- Outcomes (using ^133^Xenon and SPECT): - Increased global CBF (52.3 to 58.6 ml/100 g/min) - Reduced cerebral vascular resistance - Reduced oxygen delivery - Increased ischemic brain volume

Muench and colleagues [163]	10 SAH	Prospective interventional	Hb = 10.6 g/dl P_bt_O_2 _= 24.8 mmHg	Volume expansion with HES ± crystalloid to achieve ITBVI >1000 ml/m^2^; this produced a decline in Hb of 1.3 to 2.0 g/dl (on various days)	- Although hypervolemia/hemodilution produced a slight increment in CBF, P_bt_O_2 _decreased by an average of 0 to 5 mmHg - Only induced hypertension was consistently effective at raising P_bt_O_2_

* Dhar and colleagues [164]	8 SAH	Prospective interventional	Hb = 8.7 g/dl	One unit RBCs (mean Hb increased to 9.9 g/dl)	- Outcomes assessed using PET: - No significant change in CBF - Reduced O_2 _extraction ratio (49 to 41%; *P *= 0.06) - No significant change in CMRO_2_ - Reduction in oxygen extraction ratio observed also in territories with vasospasm and low oxygen delivery

Oddo and colleagues [165]	20 SAH	Retrospective (prospective database)	Not applicable	None	- Hb <9 g/dl associated with higher risk of P_bt_O_2 _<20 mmHg (OR 7.2, *P *< 0.01) and LPR >40 (OR 4.2, *P *= 0.02)

Chang and colleagues [237]	27 TBI	Retrospective	Not applicable	None	- 13.7% of P_bt_O_2 _readings <20 mmHg - No significant association between P_bt_O_2 _and Hb

Naidech and colleagues [238]	6 SAH	Prospective observational	Not reported	14 RBC transfusions (no protocol)	- Hb correlated with cerebral oximetry (rO_2_) - rO_2 _increased following 11/14 transfusions, but not statistically significant

Sahuquillo and colleagues [239]	28 TBI	Prospective	Not applicable	None	- Critical LOI (suggestive of ischemia/infarction) associated with lower Hb (11.7 g/dl vs. 13.1 g/dl)

Cruz and colleagues [240]	62 TBI	Retrospective (prospective data)	Not applicable	None	- Cerebral extraction of oxygen was highest when Hb <10 g/dl

* published only as abstract.

CBF = cerebral blood flow; CMRO_2 _= cerebral metabolic rate; Hb = hemoglobin; HES = hydroxyethyl starch; ITBVI = intrathoracic blood volume index; LOI = jugular venous lactate:oxygen index; LPR = lactate:pyruvate ratio; P_bt_O_2 _= brain tissue oxygen tension; PET = positron emission tomography; RBC = red blood cell; RCT = randomized controlled trial; rO_2 _= cerebral oximetry; SAH = subarachnoid hemorrhage; S_jv_O_2 _= jugular venous oxygen saturation; SPECT = single photon emission computed tomography; TBI = traumatic brain injury; TCD = transcranial Doppler.

In a retrospective study of 169 patients with TBI, Carlson and colleagues found nadir hematocrit levels to be associated with a worse Glasgow Outcome Scale at hospital discharge. However, the association between RBC transfusion and poor outcome was even stronger [143]. Other observational studies have reached similar conclusions (Table 4) [144-151]. Unfortunately, there are no large RCTs to guide practice at this time. The TRICC trial enrolled only 67 patients with severe TBI [150]. Although no statistically significant benefit from a liberal transfusion strategy was observed, this subgroup was too small to reach meaningful conclusions. Thus, the optimal use of RBCs in patients with severe TBI remains unclear. A recent survey found that practice across the USA is variable, and that the majority of clinicians believe a threshold of 7 g/dl to be too restrictive, especially in the presence of intracranial hypertension [27].

**Table 4:** Clinical studies assessing the association between hemoglobin concentrations, anemia, or transfusion and subsequent outcomes among patients with traumatic brain injury

**Study**	**Patients**	**Design and setting**	**Exposure**	**Pre-transfusion Hb or Hct**	**Analysis (variables)**	**Main result**
Carlson and colleagues [143]	169	Retrospective Single-center	- Number of days hct <30% - Nadir hct - RBC transfusion	Not reported	Linear regression assessing GOS as continuous variable	- Number of RBC units, lowest hct associated with worse discharge outcome - Number of days hct <30% associated with better outcome

‡Steyerberg and colleagues [144]	3554	*Post hoc *analysis of several RCTs Multi-center	Admission Hb (median 12.7 g/dl)	Not relevant	Logistic regression (10 covariates)	- Lower Hb associated with poor 3 to 6 month outcome (OR for 14.3 g/dl vs. 10.8 g/dl = 0.78, 0.70 to 0.87) - Laboratory variables (Hb and glucose) improved prognostic models

Duane and colleagues [145]	788	Retrospective Single-center	Hb in first 72 hours RBC transfusion	Not reported	Logistic regression (age, ISS, total blood products)	- Minimum hemoglobin in first 72 hours associated with hospital mortality (OR = 0.86, 0.73 to 1.0 per g/dl increment) - RBC transfusions not associated with mortality, but with nosocomial infection

Salim and colleagues [146]	1150	Retrospective (prospective database) Single-center	Anemia (Hb <9 g/dl; occurred in 46%) and RBC transfusion (46%)	Not reported	Logistic regression (10 covariates)	- RBC transfusion associated with hospital mortality (OR = 2.2, *P *= 0.004) and complications (OR = 3.7, *P *< 0.0001) - Anemia associated with adverse outcomes only when transfusion not included in model

George and colleagues [147]	82 (Hb 8.0 to 10.0 g/dl)	Retrospective Single-center	RBC transfusion (52%)	8.6 g/dl	Cox proportional hazard regression (age, motor GCS, blood ethanol, lowest Na^+^, complications)	RBC transfusion predicted mortality (*P *< 0.05)

‡Van Beek and colleagues [148]	3872	*Post hoc *analysis of several RCTs Multi-center	Admission Hb	Not relevant	Logistic regression (age, motor score, pupil reactivity)	- Lower Hb associated with higher risk of death/vegetative state at 3 to 6 months (OR = 0.69, 0.60 to 0.81, for 75^th ^percentile vs. 25^th ^percentile)

Schirmer-Makalsen and colleagues [149]	133	Retrospective Single-center	Hb ever <8 g/dl (22%)	Not reported	Logistic regression (10 covariates)	A single Hb <8 g/dl did not predict adverse outcome

McIntyre and colleagues [150]	67	*Post hoc *analysis of RCT Multi-center	Comparison of transfusion thresholds of 7.0 g/dl vs. 10.0 g/dl	Not reported	Logistic regression (age, APACHE II, PAC use)	- 30-day mortality 17% in restrictive group vs. 13% in liberal group (*P *= 0.64) - Development of MOD and ICU LOS similar in both groups

Robertson and colleagues [151]	102	Prospective Single-center	Hb at time of CBF determination	Not reported	Logistic regression (age, CBF, GCS, CPP, CMRO_2_)	- Lower Hb associated with unfavorable GOS after 3 months

‡ Based, in part, on same datasets

APACHE = Acute Physiology and Chronic Health Evaluation; CBF = cerebral blood flow; CMRO_2 _= cerebral metabolic rate; CPP = cerebral perfusion pressure; GCS = Glasgow Coma Scale; GOS = Glasgow Outcome Scale; Hb = hemoglobin; hct = hematocrit; ICU = intensive care unit; ISS = injury severity score; LOS = length of stay; MOD = multiple organ dysfunction; OR = odds ratio; PAC = pulmonary artery catheter; RBC = red blood cell; RCT = randomized controlled trial.

#### Subarachnoid hemorrhage

Narrowing of the cerebral vasculature (angiographic vasospasm) complicates about two-thirds of cases of SAH. Vasospasm most often emerges between days 3 and 14 after SAH and is the most important cause of secondary brain injury [87]. Evidence of cerebral infarction that was not present initially is observed in as many as 50 to 70% of survivors using magnetic resonance imaging (MRI) [152,153]. Unlike other forms of stroke, the predictable risk of vasospasm and cerebral ischemia provides a unique opportunity for the provision of neuroprotection prior to the insult.

Three studies have assessed the association between daily Hb concentrations and eventual neurologic outcome [154-156]. Each of these demonstrated that patients with an unfavorable outcome consistently have lower Hb levels throughout much of the first two weeks in hospital (Table 5). The degree of decrement in Hb levels over time was also highly predictive of outcome [154]. Despite the use of multivariable analyses, there were numerous potentially confounding variables that could not be adjusted for. For example, patients who are 'sicker' tend to have more blood drawn for laboratory tests, have more invasive procedures performed, and tend to receive more intravenous fluids, all of which could contribute to lower Hb concentrations. Thus, the association between lower Hb and poor outcome has not conclusively been proven to be causative.

**Table 5:** Clinical studies assessing the association between hemoglobin concentrations, anemia, or transfusion and subsequent outcomes among patients with aneurysmal subarachnoid hemorrhage

Study	Patients	Design and setting	Exposure	Mean pre-transfusion Hb/Hct	Analysis (variables)	Main result
‡Kramer and colleagues [28]	245	Retrospective Single-center	- Anemia (nadir Hb <10 g/dl) - RBC transfusion (35%)	9.5 g/dl No transfusion protocol	Logistic regression (WFNS score, age, vasospasm, modified Fisher score)	- Anemia and transfusion associated with poor 6 week outcome (association stronger for transfusion) - RBCs associated with nosocomial infection - Age of blood not associated with complications

‡Kramer and colleagues [154]	245	Retrospective Single-center	Daily nadir Hb over 2 weeks	9.5 g/dl No transfusion protocol	GEE to account for correlated data (WFNS score, age, vasospasm, modified Fisher score)	- Hb and decline in Hb over time predict poor outcome - Association between Hb and outcome stronger among high grade patients

†Naidech and colleagues [155]	611	Retrospective (prospective database) Single-center	- Mean and nadir Hb over 2 weeks - 35% transfused	Not reported No transfusion protocol	Multinomial regression (Hunt-Hess, age, cerebral infarction)	Higher nadir (but not mean) Hb associated with better outcome after 3 months (OR = 0.83 per 10 g/dl increase; *P *= 0.04)

Naidech and colleagues [156]	103	Retrospective (prospective database) Single-center	- Mean Hb over 2 weeks - 47% transfused	9.2 g/dl No transfusion protocol	Logistic regression (Hunt-Hess, age, angiographic vasospasm)	Higher 2 week mean Hb associated with better outcome at discharge (OR = 0.57 per 10 g/dl increase; *P *= 0.04)

Tseng and colleagues [157]	160	*Post hoc *analysis 2 RCTs) Single-center	RBC transfusion (19%)	Not reported	Logistic regression (age, WFNS, IVH, postoperative deficits, sepsis, DIDs)	- Transfusion associated with poor outcome at discharge (OR = 4.5, *P *= 0.04) but not 6 months - More colloid use predicted lower hct and need for transfusion

†Wartenberg and colleagues [158]	576	Retrospective (prospective database) Single-center	Anemia (Hb <9 g/dl treated with transfusion; 36% of cohort)	Not reported No transfusion protocol	Logistic regression (Hunt-Hess, age, cerebral infarction, re-bleeding, aneurysm size >10 mm)	Anemia associated with worse 3 month outcome (OR = 1.8; *P *= 0.02)

* DeGeorgia and colleagues [159]	166	Retrospective Single-center	RBC Transfusion (49%)	Not reported No transfusion protocol	Logistic regression (Hunt-Hess, APACHE II)	Transfusion associated with worse outcome at discharge among patients with vasospasm, not without (OR = 2.9, CI = 1.1 to 7.8)

Smith and colleagues [160]	441	Retrospective (prospective database) Single-center	RBC transfusion (61%)	Intra-operative: 39.6% Post-operative: 32.0% No transfusion protocol	Logistic regression (Hunt-Hess, Fisher, smoking, intra-operative rupture, delay to surgery)	- Intraoperative transfusion associated with poor 6 month outcome (OR = 2.4, CI = 1.3 to 4.5) - Postoperative transfusion associated with angiographic vasospasm (OR = 1.7, CI = 1.0 to 2.8))

‡ & †: studies used same datasets; *: published only as abstract

APACHE = Acute Physiology and Chronic Health Evaluation; CI = 95% confidence intervals; DID = delayed ischemic deficit; GEE = generalized estimating equation; Hb = hemoglobin; hct = hematocrit; IVH = intraventricular hemorrhage; OR = odds ratio; RBC = red blood cell; RCT = randomized controlled trial; WFNS = World Federation of Neurological Surgeons score.

As in other settings, several studies have also shown a strong association between transfusion and unfavorable outcomes following SAH (Table 5) [28,157-160]. One unconfirmed report suggested that the use of RBCs could contribute to the development of cerebral vasospasm, perhaps by promoting inflammation or depleting endogenous NO supplies [160]. A recent observational study found no difference in complications based on the transfusion of older (>21 days) compared with newer (≤21 days) units of blood, although this assessment was based on only 85 transfused patients [28].

Hemodilution, together with hypervolemia and hypertension, has been used as part of 'triple H therapy', a therapeutic strategy to improve CBF in patients with vasospasm [161]. One study used ^133^Xenon injections to assess global CBF in eight patients with SAH. As expected, isovolemic hemodilution from a mean Hb of 11.9 to 9.2 g/dl produced an increase in global CBF and a reduction in cerebral vascular resistance. However, the increase in CBF was not sufficient to overcome the reduction in C_a_O_2_, such that global O_2 _delivery fell and ischemic brain volume actually increased [162]. Complimentary findings were subsequently reported by Muench and colleagues, who used aggressive volume expansion on days 1, 3, and 7, which produced a concomitant reduction in Hb concentration ranging from of 1.3 to 2.0 g/dl. Although this intervention consistently produced a small increment in CBF, it actually caused a proportionally larger decline in P_bt_O_2 _(Table 3) [163].

More recently, Dhar and colleagues assessed the effects of transfusion in patients with SAH using PET [164]. PET scans were performed before and after the administration of one unit of RBCs to patients with pre-transfusion Hb concentrations less than 10 g/dl. Although no change in CMRO_2 _was observed, OEF dropped from 49 to 41%. Thus, it is possible that in vulnerable regions of the brain with relatively high OEF, RBC transfusions could help avoid irreversible infarction. Another recent study of 20 SAH patients found Hb concentrations less than 9 g/dl to be associated with lower P_bt_O_2 _and higher LPR [165].

In summary, there is now extensive data to suggest that even moderate degrees of anemia are associated with worse physiologic parameters and clinical outcomes in patients with SAH. However, it is not clear that the use of RBC transfusions can modify these associations. An adequately powered, RCT comparing different transfusion thresholds is urgently required, especially in light of the vulnerability of these patients to delayed cerebral ischemia and the frequency with which they develop anemia.

#### Ischemic stroke

Because of the known inverse relation between hematocrit and CBF, there has long been interest in the clinical use of hemodilution in the management of acute ischemic stroke [166]. Some studies have suggested that relatively high Hb concentrations may predispose to the development of strokes [167-173], as well as contribute to worse outcomes when cerebral ischemia occurs [174-177]. It is conceivable that increased viscosity could have a particularly deleterious effect on microvascular flow through the ischemic penumbra. Consistent with this notion, Allport and colleagues performed serial MRI scans in 64 stroke patients and found that a higher baseline hematocrit was independently associated with infarct growth and less chance of successful reperfusion [178].

The deleterious association with a higher hematocrit has, however, been inconsistent and largely observed at levels in excess of 45% (Table 6). Indeed, several studies have shown a U-shaped relation where low hematocrit levels are also associated with larger infarct size and worse outcomes [175,177,179-184]. The lowest risk of stroke and the best outcomes have generally been observed with mid-range hematocrit levels of about 42 to 45% [172,175]. This range was also supported by a study using ^133^Xe to assess CBF in stroke patients, with the finding that cerebral O_2 _delivery was optimized at a hematocrit level of 40 to 45% [185]. Conversely, several animal studies have suggested that cerebral O_2 _delivery and neuroprotection are optimized at slightly lower hematocrit or Hb values, in the range of 30 to 36% and 10 to 12 g/dl, respectively [58,186,187]. Greater degrees of hemodilution consistently appear to be deleterious [188]. Some case reports have even described patients with relatively stenotic cerebral vessels who may have developed ischemic strokes directly attributable to anemia [189-191].

**Table 6:** Studies assessing the association between hemoglobin concentrations or anemia and subsequent clinical outcomes among patients with acute ischemic stroke

**Study**	**Patients**	**Design and setting**	**Exposure**	**Outcome**	**Main result**	**Comment**
Sacco and colleagues [174]	3481 ischemic stroke	Retrospective (prospective data-base) Multi-center	Baseline hct (patients divided into quartiles)	Death at 28 days	Hct >46% associated with death, but only among women	Hct ≤40% represented lowest quartile; effects of more extreme anemia not reported

Diamond and colleagues [175]	1012 ischemic stroke	Retrospective Single-center	Baseline hct Median 41%; inter-quartile range 38 to 44%	Discharge home (rather than nursing facility)	High and low hct associated with worse outcome (U shaped curve) Optimal hct 45%	Only 2% of patients had hct <30% at time of their stroke

Lowe and colleagues [177]	270 ischemic stroke	Retrospective Single-center	Baseline hct	Death in hospital	Patients with high hct (≥50%) had higher mortality (66 to 71%)	Elderly (≥75) with hct <40% also had higher mortality (65%)

Allport and colleagues [178]	64 hemispheric ischemic stroke	Prospective Single-center	Baseline hct Median 42%; range 33 to 48%	Reperfusion, infarct growth on serial MRI	Higher hct associated with less reperfusion (OR = 0.53, *P *< 0.0001) and more infarct growth (OR = 1.26, *P *< 0.05)	This was a study of the effects of high hct; few patients were anemic

†Huang and colleagues [179]	774 ischemic stroke	Prospective Single-center	Anemia (Hb <13 g/dl for men, <12 g/dl for women) (21%)	Death and mRS ≥3 at 3 years	Anemic patients more likely to die (OR = 2.2, *P *= 0.02) and to have a poor neurological outcome (67% vs. 60%, *P *= 0.07)	Numerous potential confounders not adjusted for; severity of anemia not well characterized

†Huang and colleagues [180]	66 ischemic stroke (complicating ICA occlusion)	Prospective Single center	Anemia (Hb <13 for men, <12 for women)	Death or recurrent stroke at 2 years	Anemia associated with death or recurrent stroke at 2 years (OR = 5.1, *P *= 0.012)	Numerous potential confounders not adjusted for; severity of anemia not well characterized

Nybo and colleagues [181]	250 ischemic stroke	Retrospective Single-center	Anemia (Hb <13 g/dl for men, <12 g/dl for women) (15%)	Death at 6 months	Anemia associated with greater risk of death (OR = 3.6, CI = 1.4 to 9.3)	Numerous potential confounders not adjusted for; severity of anemia not well characterized

Bhatia and colleagues [182]	116 ischemic or hemorrhagic stroke	Retrospective Single-center	Baseline Hb	Death at 30 days	Hb not associated with risk of death	Degree of anemia relatively mild

Wade and colleagues [183]	1377 symptomatic cerebrovascular disease	Retrospective (*post hoc *review of prospective RCT) Multi-center	Hb >15 g/dl vs. ≥15 g/dl at study entry	Stroke	Patients with Hb ≥15 had similar outcomes to patients with Hb <15 g/dl	This was a study of the effects of high Hb; few patients were anemic

LaRue and colleagues [184]	2077 ischemic or hemorrhagic stroke	Retrospective (prospective database) Multi-center	Baseline hct (patients divided into quartiles)	Death in hospital	Hct not predictive of death (neither when high nor low)	Neurologic outcomes (other than death) not reported

CI = confidence interval; Hb = hemoglobin; hct = hematocrit; ICA = internal carotid artery; MRI = magnetic resonance imaging; mRS = modified Rankin scale; OR = odds ratio; RCT = randomized controlled trial

Several RCTs and a meta-analysis have not shown any clear benefit to using hemodilution as a therapeutic strategy in acute ischemic stroke [192]. However, there was a great deal of heterogeneity in the methodology of these studies (timing of treatment, specific type and dose of plasma expander, target hematocrit). Although each study deliberately produced reductions in hematocrit with the use of colloids and/or phlebotomy, the reductions were relatively modest, generally not beyond 37 to 38% [192-196].

More recently, several animal studies and phase II human trials have suggested that hemodilution with relatively high doses of albumin may reduce infarct size and enhance the efficacy of thrombolytic therapy [197-200]. It is likely that this effect was observed, in part, because of the unique properties of albumin, rather than only hemodilution. In a phase II dose-finding study, the reduction in hematocrit induced by the highest doses of albumin averaged 6 to 10% [198,199].

In summary, there is currently no routine role for hemodilution in the management of acute ischemic stroke. Whether transfusing anemic stroke patients with Hb concentrations lower than 9 to 11 g/dl is beneficial has not been well evaluated.

#### Intracerebral hemorrhage

There has been controversy regarding the importance of cerebral ischemia in causing secondary brain injury after ICH. Early studies had suggested that an expanding intracerebral hematoma could cause mechanical compression and vasoconstriction of the surrounding vasculature, thereby producing a 'perihematomal penumbra' [201-203]. Imaging with PET, CT perfusion scans, and MRI have confirmed that the majority of patients with ICH have a surrounding rim of hypoperfusion [91,204-206]. The biochemistry of this region appears to be similar to that of traumatic cerebral contusions [207]. However, OEF is not increased in the perihematomal tissues, suggesting that this hypoperfusion is due to reduced cerebral metabolism, rather than true ischemia [91]. Thus, mild reductions in Hb concentration are unlikely to have a major impact in contributing to neuronal death. Nevertheless, it remains uncertain whether perihematomal tissues tolerate anemia as well as healthy brain.

#### Use of hemoglobin-based blood substitutes

Hb-based blood substitutes (HBBS) have theoretical advantages over other fluids in the resuscitation of neurocritical care patients, because they have the potential to achieve the CBF-enhancing effects of hemodilution, while concomitantly maintaining, or even raising, C_a_O_2_. Several animal studies performed in the setting of experimental ischemic stroke, TBI, and SAH-induced vasospasm have supported this concept [208-221]. Alternatively, free Hb may also have numerous deleterious effects, probably mediated, in large part, by scavenging of NO [222]. Although not all products are identical, a recent meta-analysis of RCTs suggested that their use is associated with an increased risk of death and myocardial infarction [223]. One phase II RCT involving 85 patients with ischemic stroke reported worse neurological outcomes with the use of diaspirin cross-linked Hb [224]. Of the five RCTs involving trauma patients, none specifically assessed the subgroup of patients with TBI, although the largest study reported no statistically significant interaction between HBBS and admission Glasgow coma scale on mortality [225-229]. Two of the three RCTs in the setting of cardiac surgery reported the occurrence of perioperative stroke; there were no differences between HBBS-treated and control patients [230,231]. Thus, although the use of HBSS in neurocritical care should be further investigated, there is currently no role for the routine use of these products.

## Conclusions

Anemia is common in neurocritical care patients, is associated with worse outcomes, and should be avoided as much as possible with blood conservation strategies. Although Hb concentrations as low as 7 g/dl are well tolerated by most critically ill patients [25], there is ample data from animal studies, as well as human physiologic and observational studies to suggest that such a severe degree of anemia could be harmful in the brain-injured patient. Thus, in our practice, we frequently transfuse selected patients with Hb concentrations less than 8 to 9 g/dl. However, because allogeneic RBCs have multiple potentially deleterious effects, it cannot be assumed that the use of transfusions to 'correct' Hb levels alters the association between anemia and adverse outcomes. The impact of the duration of blood storage on the neurologic implications of transfusion requires further investigation. Unfortunately, existing guidelines provide little guidance to clinicians in deciding when to transfuse anemic stroke and neurocritical care patients [232-236]; clearly, RCTs are needed.

## Key messages

• Despite an increment in cerebral blood flow, even moderate reductions in Hb concentration lead to less overall cerebral oxygen delivery, resulting in lower P_bt_O_2 _and 'metabolic distress' (higher OEF and LPR).

• Although the relation has not been proven with certainty to be causative, anemia is consistently associated with worse outcomes among neurocritical care patients.

• Despite some beneficial physiologic effects (increased P_bt_O_2 _and reduced OEF), it remains uncertain whether transfusion can improve cerebral metabolism and help salvage tenuous 'penumbral' brain tissue, thereby improving neurologic recovery.

• Although a transfusion threshold of 7 g/dl is safe in many general critical care patients, it remains unclear if this is also true in neurocritical care patients.

• The duration of red blood cell storage may have implications on the cerebral consequences of transfusion.

## Abbreviations

CBF: cerebral blood flow; C_a_O_2_: arterial oxygen content; CMRO_2_: cerebral metabolic rate; CO: cardiac output; CO_2_: carbon dioxide; CPP: cerebral perfusion pressure; DO_2_: oxygen delivery; Hb: hemoglobin; HBBS: hemoglobin-based blood substitutes; ICH: intracerebral hemorrhage; ICU: intensive care unit; LPR: lactate to pyruvate ratio; MRI: magnetic resonance imaging; NO: nitric oxide; O_2_: oxygen; OEF: oxygen extraction fraction; P_bt_O_2_: brain tissue oxygen tension; PCO_2_: partial pressure of carbon dioxide; PET: positron emission tomography; PO_2_: partial pressure of oxygen; RBC: red blood cell; RCT: randomized controlled trial; SAH: subarachnoid hemorrhage; SaO_2_: oxygen saturation; S_jv_O_2_: jugular venous oxygen saturation; TBI: traumatic brain injury.

## Competing interests

The authors declare that they have no competing interests.

## Authors' contributions

AHK was responsible for the conception and design of the study, the analysis and interpretation of the data, and the drafting and revision of the manuscript. DAZ was responsible for the analysis and interpretation of data, and the revision of the manuscript. Both authors approved the final version of the manuscript.

## References

[B1] Al Thanayan E, Bolton C, Hichici D, Savard M, Teitelbaum J, Young B, Zygun D (2008). Neurocritical care in Canada: evolving streams in a new discipline. Can J Neurol Sci.

[B2] Elf K, Nilsson P, Enblad P (2002). Outcome after traumatic brain injury improved by an organized secondary insult program and standardized neurointensive care. Crit Care Med.

[B3] Patel HC, Menon DK, Tebbs S, Hawker R, Hutchinson PJ, Kirkpatrick P (2002). Specialist neurocritical care and outcome from head injury. Intensive Care Med.

[B4] Miller JD, Sweet RC, Narayan R, Becker DP (1978). Early insults to the injured brain. JAMA.

[B5] Chesnut RM, Marshall LF, Klauber MR, Blunt BA, Baldwin N, Eisenberg HM, Jane JA, Marmarou A, Foulkes MA (1993). The role of secondary brain injury in determining outcome from severe head injury. J Trauma.

[B6] McHugh GS, Engel DC, Butcher I, Steyerberg EW, Lu J, Mushkudiani N, Hernandez AV, Marmarous A, Maas AI, Murray GD (2007). Prognostic value of secondary insults in traumatic brain injury: results from the IMPACT study. J Neurotrauma.

[B7] Henzler D, Cooper DJ, Tremayne AB, Rossaint R, Higgins A (2007). Early modifiable factors associated with fatal outcome in patients with severe traumatic brain injury: a case control study. Crit Care Med.

[B8] Iron Deficiency Anemia Assessment, Prevention, and Control: A guide for programme managers. http://whqlibdoc.who.int/hq/2001/WHO_NHD_01.3.pdf.

[B9] Rodriguez RM, Corwin HL, Gettinger A, Corwin MJ, Gubler D, Pearl RG (2001). Nutritional deficiencies and blunted erythropoietin response as causes of anemia of critical illness. J Crit Care.

[B10] Vincent JL, Baron JF, Reinhart K, Gattinoni L, Thijs L, Webb A, Meier-Hellmann A, Nollet G, Peres-Bota D (2002). Anemia and blood transfusion in critically ill patients. JAMA.

[B11] Corwin HL, Gettinger A, Pearl RG, Fink MP, Levy MM, Abraham E, MacIntyre NR, Shabot MM, Duh MS, Shapiro MJ (2004). The CRIT Study: Anemia and blood transfusion in the critically ill – current practice in the United States. Crit Care Med.

[B12] Nguyen BV, Bota DP, Melot C, Vincent JL (2003). Time course of hemoglobin concentrations in nonbleeding intensive care unit patients. Crit Care Med.

[B13] Scharte M, Fink MP (2003). Red blood cell physiology in critical illness. Crit Care Med.

[B14] Rogiers P, Zhang H, Leeman M, Nagler J, Neels H, Mélot C, Vincent JL (1997). Erythropoietin response in blunted in critically ill patients. Intensive Care Med.

[B15] van Iperen CE, Gaillard CA, Kraaijenhagen RJ, Braam BG, Marx JJ, Wiel A van de (2000). Response of erythropoiesis and iron metabolism to recombinant human erythropoietin in intensive care unit patients. Crit Care Med.

[B16] Darveau M, Denault AY, Blais N, Notebaert E (2004). Bench-to-bedside review: iron metabolism in critically ill patients. Crit Care.

[B17] Corwin HL, Krantz SB (2000). Anemia of the critically ill: "Acute" anemia of chronic disease. Crit Care Med.

[B18] Walsh TS, Saleh EE (2006). Anemia during critical illness. Br J Anesth.

[B19] Smoller BR, Kruskall MS (1986). Phlebotomy for diagnostic laboratory tests in adults. Patterns of use and effect on transfusion requirements. N Engl J Med.

[B20] Tarpey J, Lawler PG (1990). Iatrogenic anemia? A survey of venesection in patients in the intensive therapy unit. Anaesthesia.

[B21] Zimmerman JE, Seneff MG, Sn X, Wagner DP, Knaus WA (1997). Evaluating laboratory usage in the intensive care unit: patient and institutional characteristics that influence frequency of blood sampling. Crit Care Med.

[B22] Berezina TL, Zaets SB, Kozhura VL, Novoderzhkina IS, Kirsanova AK, Deitch EA, Machiedo GW (2001). Morphologic changes of red blood cells during hemorrhagic shock replicate changes of aging. Shock.

[B23] Walsh TS, Garrioch M, Maciver C, Lee RJ, MacKirdy F, McClelland DB, Kinsella J, Wallis C (2004). Red cell requirements for intensive care units adhering to evidence-based transfusion guidelines. Transfusion.

[B24] French CJ, Bellomo R, Finfer SR, Lipman J, Chapman M, Boyce NW (2002). Appropriateness of red blood cell transfusion in Australasian intensive care practice. Med J Aust.

[B25] Hebert PC, Wells G, Blajchman MA, Marshall J, Martin C, Pagliarello G, Tweeddale M, Schweitzer I, Yetisir E (1999). A multicenter, randomized, controlled clinical trial of transfusion requirements in critical care. Transfusion Requirements in Critical Care Investigators, Canadian Critical Care Trials Group. N Engl J Med.

[B26] Lacroix J, Hebert PC, Hutchison JS, Hume HA, Tucci M, Ducruet T, Gauvin F, Collet JP, Toledano BJ, Robillard P, Joffe A, Biarent D, Meert K, Peters MJ (2007). TRIPICU Investigators; Canadian Critical Care Trials Group; Pediatric Acute Lung Injury and Sepsis Investigators Network. Transfusion strategies for patients in pediatric intensive care units. N Engl J Med.

[B27] Sena MJ, Rivers RM, Muizelaar JP, Battistella FD, Utter GH (2009). Transfusion practices for acute traumatic brain injury: a survey of physicians at US trauma centers. Intensive Care Med.

[B28] Kramer AH, Gurka MJ, Nathan B, Dumont AS, Kassell NF, Bleck TP (2008). Complications associated with anemia and blood transfusion in patients with aneurismal subarachnoid hemorrhage. Crit Care Med.

[B29] Pendem S, Rana S, Manno EM, Gajic O (2006). A review of red cell transfusion in the neurological intensive care unit. NeuroCrit Care.

[B30] Vavilala MS, Lee LA, Lam AM (2002). Cerebral blood flow and vascular physiology. Anesthesiol Clin North America.

[B31] Marshall RS (2004). The functional relevance of cerebral hemodynamics: why blood flow matters to the injured and recovering brain. Curr Opin Neurol.

[B32] Udomphorn Y, Armstead WM, Vavilala MS (2008). Cerebral blood flow and autoregulation after pediatric traumatic brain injury. Pediatr Neurol.

[B33] Hatcher JD, Chiu LK, Jennings DB (1978). Anemia as a stimulus to aortic and carotid chemoreceptors in the cat. J Appl Physiol.

[B34] Chapler CK, Cain SM (1985). The physiologic reserve in oxygen carrying capacity: studies in experimental hemodilution. Can J Physiol Pharmacol.

[B35] Habler OP, Kleen MS, Podtschaske AH, Hutter JW, Tiede M, Kemming GI, Welte MV, Corso CO, Messmer KF (1996). The effect of acute normovolemic hemodilution (ANH) on myocardial contractility in anesthetized dogs. Anesth Analg.

[B36] Murray JF, Escobar E, Rapaport E (1969). Effects of blood viscosity on hemodynamic responses in acute normovolemic anemia. Am J Physiol.

[B37] Fowler NO, Holmes JC (1975). Blood viscosity and cardiac output in acute experimental anemia. J Appl Physiol.

[B38] Hebert PC, Linden P Van der, Biro G, Hu LQ (2004). Physiologic aspects of anemia. Crit Care Clin.

[B39] Vespa PM (2006). The implications of cerebral ischemia and metabolic dysfunction for treatment strategies in neurointensive care. Curr Opin Crit Care.

[B40] Vespa P, Bergsneider M, Hattori N, Wu HM, Huang SC, Martin NA, Glenn TC, McArthur DL, Hovda DA (2005). Metabolic crisis without brain ischemia is common after traumatic brain injury: a combined microdialysis and positron emission tomography study. J Cereb Blood Flow Metab.

[B41] Senda M, Alpert NM, Mackay BC, Buxton RB, Correia JA, Weise SB, Ackerman RH, Dorer D, Buonanno FS (1989). Evaluation of the ^11 ^CO_2 _positron emission tomographic method for measuring brain pH. II. Quantitative pH mapping in patients with ischemic cerebrovascular diseases. J Cereb Blood Flow Metab.

[B42] Coles JP, Fryer TD, Smielewski P, Rice K, Clark JC, Pickard JD, Menon DK (2004). Defining ischemic burden after traumatic brain injury using ^15^O PET imaging of cerebral physiology. J Cereb Blood Flow Metab.

[B43] Sakoh M, Ostergaard L, Rohl L, Smith DF, Simonsen CZ, Sorensen JC, Poulsen PV, Gyldensted C, Sakaki S, Gjedde A (2000). Relationship between residual cerebral blood flow and oxygen metabolism as predictive of ischemic tissue viability: sequential multitracer positron emission tomography scanning of middle cerebral artery occlusion during the critical first 6 hours after stroke in pigs. J Neurosurg.

[B44] Weiskopf RB, Viele MK, Feiner J, Kelley S, Liberman J, Noorani M, Leung JM, Fisher DM, Murray WR, Toy P, Moore MA (1998). Human cardiovascular and metabolic response to acute, severe isovolemic anemia. JAMA.

[B45] Kothaveale A, Banki NM, Kopelnik A, Yarlagadda S, Lawton MT, Ko N, Smith WS, Drew B, Foster E, Zaroff JG (2006). Predictors of left ventricular regional wall motion abnormalities after subarachnoid hemorrhage. Neurocrit Care.

[B46] Hare GMT, Tsui AKY, McLaren AT, Ragoonanan TE, Yu J, Mazer CD (2008). Anemia and cerebral outcomes: Many questions, fewer answers. Anesth & Analg.

[B47] Bruder N, Cohen B, Pellissier D, Francois G (1998). The effect of hemodilution on cerebral blood flow velocity in anesthetized patients. Anesth Analg.

[B48] Rebel A, Ulatowski JA, Kwansa H, Bucci E, Koehler RC (2003). Cerebrovascular response to decreased hematocrit: effect of cell-free hemoglobin, plasma viscosity, and CO_2_. Am J Physiol Heart Circ Physiol.

[B49] van Bommel J, Trouwborst A, Schwarte L, Siegemund M, Ince C, Henny C (2002). Intestinal and cerebral oxygenation during severe isovolemic hemodilution and subsequent hyperoxic ventilation in a pig model. Anesthesiology.

[B50] Hudetz AG, Wood JD, Kampine JP (2000). 7-Nitroindazole impedes erythrocyte flow response to isovolemic hemodilution in the cerebral capillary circulation. J Cereb Blood Flow Metab.

[B51] Hudetz AG, Shen H, Kampine JP (1998). Nitric oxide from neuronal NOS plays critical role in cerebral capillary flow response to hypoxia. Am J Physiol.

[B52] Hare GMT, Mazer CD, Mak W, Gorczynski RM, Hum KM, Kim SY, Wyard L, Barr A, Qu R, Baker AJ (2003). Hemodilutional anemia is associated with increased cerebral neuronal nitric oxide synthase gene expression. J Appl Physiol.

[B53] Plochl W, Liam BL, Cook DJ, Orszulak TA (1999). Cerebral response to hemodilution during cardiopulmonary bypass in dogs: the role of nitric oxide synthase. Br J Anaesth.

[B54] Ulatwoski JA, Bucci E, Nishikawa T, Razynska A, Williams MA, Takeshima R, Traystman RJ, Koehler RC (1996). Cerebral O2 transport with hematocrit reduced by cross-linked hemoglobin transfusion. Am J Physiol.

[B55] Todd MM, Farrell S, Wu B (1997). Cerebral blood flow during hypoxemia and hemodilution in rabbits: different roles for nitric oxide?. J Cereb Blood Flow Metab.

[B56] Hare GM, Worrall JM, Baker AJ, Liu E, Sikich N, Mazer CD (2006). Beta2 adrenergic antagonist inhibits cerebral cortical oxygen delivery after severe hemodilution in rats. Br J Anaesth.

[B57] McLaren AT, Marsden PA, Mazer CD, Baker AJ, Stewart DJ, Tsui AK, Li X, Yucel Y, Robb M, Boyd SR, Liu E, Yu J, Hare GM (2007). Increased expression of HIF-1alpha, nNOS, and VEGF in the cerebral cortex of anemic rats. Am J Physiol Regul Integr Comp Physiol.

[B58] Dexter F, Hindman BJ (1997). Effect of haemoglobin concentration on brain oxygenation in focal stroke: a mathematical modeling study. Br J Anaesth.

[B59] Todd MM, Wu B, Maktabi M, Hindman BJ, Warner DS (1994). Cerebral blood flow and oxygen delivery during hypoxemia and anemia: The role of arterial oxygen content. Am J Phys.

[B60] Korosue K, Heros RC (1992). Mechanism of cerebral blood flow augmentation by hemodilution in rabbits. Stroke.

[B61] Cole DJ, Drummond JC, Patel PM, Marcantonio S (1994). Effects of viscosity and oxygen content on cerebral blood flow in ischemic and normal rat brain. J Neurol Sci.

[B62] Tu YK, Kuo MF, Liu HM (1997). Cerebral oxygen transport and metabolism during graded isovolemic hemodilution in experimental global ischemia. J Neurol Sci.

[B63] Todd MM, Wu B, Warner DS (1994). The hemispheric cerebrovascular response to hemodilution is attenuated by a focal cryogenic brain injury. J Neurotrauma.

[B64] Weiskopf RB, Kramer JH, Viele M, Neumann M, Feiner JR, Watson JJ, Hopf HW, Toy P (2000). Acute severe isovolemic anemia impairs cognitive function and memory in humans. Anesthesiology.

[B65] Weiskopf RB, Toy P, Hopf HW, Feiner J, Finlay HE, Takahashi M, Bostrom A, Songster C, Aminoff MF (2005). Acute isovolemic anemia impairs central processing as determined by P300 latency. Clin Neurophysiol.

[B66] Ge YL, Lv R, Zhou W, Ma XX, Zhong TD, Duan ML (2007). Brain damage following severe acute normovolemic hemodilution in combination with controlled hypotension in rats. Acta Anaesthesiol Scand.

[B67] Lee LA, Deem S, Glenny RW, Townsend I, Moulding J, An D, Treggiari MM, Lam A (2008). Effects of anemia and hypotension on porcine optic nerve blood flow and oxygen delivery. Anesthesiology.

[B68] Czinn EA, Salem MR, Crystal GJ (1995). Hemodilution impairs hypocapnia-induced vasoconstrictor responses in the brain and spinal cord in dogs. Anesth Analg.

[B69] Tu YK, Liu HM (1996). Effects of isovolemic hemodilution on hemodynamics, cerebral perfusion, and cerebral vascular reactivity. Stroke.

[B70] Kuwabara Y, Sasaki M, Hirakata H, Koga H, Nakagawa M, Chen T, Kaneko K, Masuda K, Fujishima M (2002). Cerebral blood flow and vasodilatory capacity in anemia secondary to chronic renal failure. Kidney Int.

[B71] Tinmouth A, Fergusson D, Yee IC, Hebert PC (2006). ABLE Investigators; Canadian Critical Care Trials Group. Clinical consequences of red cell storage in the critically ill. Transfusion.

[B72] Berezina TL, Zaets SB, Morgan C, Spillert CR, Kamiyama M, Spolarics Z, Deitch EA, Machiedo GW (2002). Influence of storage on red blood cell rheological properties. J Surg Res.

[B73] Tsai AG, Cabrales P, Intaglietta M (2004). Microvascular perfusion upon exchange transfusion with stored red blood cells in normovolemic anemic conditions. Transfusion.

[B74] Heaton A, Keegan T, Holme S (1989). In vivo regeneration of red cell 2,3-diphosphoglycerate following transfusion of DPG-depleted AS-1, AS-3 and CPDA-1 red cells. Br J Haematol.

[B75] Marik PE, Sibbald WJ (1993). Effect of stored-blood transfusion on oxygen delivery in patients with sepsis. JAMA.

[B76] Walsh TS, McArdle F, McLellan SA, Maciver C, Maginnis M, Prescott RJ, McClelland DB (2004). Does the storage time of transfused red blood cells influence regional or global indexes of tissue oxygenation in anemic critically ill patients?. Crit Care Med.

[B77] Suttner S, Piper SN, Kumle B, Lang K, Rohm KD, Isgro F, Boldt J (2004). The influence of allogeneic red blood cell transfusion compared with 100% oxygen ventilation on systemic oxygen transport and skeletal muscle oxygen tension after cardiac surgery. Anesth Analg.

[B78] Gramm J, Smith S, Gamelli RL, Dries DJ (1996). Effect of transfusion on oxygen transport in critically ill patients. Shock.

[B79] Goldman M, Webert KE, Arnold DM, Freedman J, Hannon J, Blajchman MA (2005). TRALI Consensus Panel. Transfus Med Rev.

[B80] Schorr AF, Duh MS, Kelly KM, Kollef MH (2004). Red blood cell transfusion and ventilator-associated pneumonia: A potential link?. Crit Care Med.

[B81] Gunst MA, Minei JP (2007). Transfusion of blood products and nosocomial infection in surgical patients. Curr Opin Crit Care.

[B82] Gong MN, Thompson BT, Williams P, Pothier L, Boyce PD, Christiani DC (2005). Clinical predictors of and mortality in acute respiratory distress syndrome: potential role of red cell transfusion. Crit Care Med.

[B83] Silverboard H, Aisiku I, Martin GS, Adams M, Rozycki G, Moss M (2005). The role of acute blood transfusion in the development of acute respiratory distress syndrome in patients with severe trauma. J Trauma.

[B84] Hebert PC, Fergusson D, Blajchman MA, Wells GA, Kmetic A, Coyle D, Heddle N, Germain M, Goldman M, Toye B, Schweitzer I, vanWalraven C, Devine D, Sher GD (2003). Clinical outcomes following institution of the Canadian universal leukoreduction program for red blood cell transfusions. JAMA.

[B85] Rigamonti A, McLaren AT, Mazer CD, Nix K, Ragoonanan T, Freedman J, Harringon A, Hare GM (2008). Storage of strain-specific rat blood limits cerebral tissue oxygen delivery during acute fluid resuscitation. Br J Anaesth.

[B86] Weiskopf RB, Kramer JH, Viele M, Neumann M, Feiner JR, Watson JJ, Hopf HW, Toy P (2000). Acute severe isovolemic anemia impairs cognitive function and memory in humans. Anesthesiology.

[B87] Kassell NF, Torner JC, Haley EC, Jane JA, Adams HP, Kongable GL (1990). The International Cooperative Study on the Timing of Aneurysm Surgery. Part 1: Overall management results. J Neurosurg.

[B88] Dorsch NW, King MT (1994). A review of cerebral vasospasm in aneurysmal subarachnoid hemorrhage. Part 1: Incidence and effects. J Clin Neurosci.

[B89] Diringer MN, Yundt K, Videen TO, Adams RE, Zazulia AR, Deibert E, Aiyagari V, Dacey RG, Grubb RL, Powers WJ (2000). No reduction in cerebral metabolism as a result of early moderate hyperventilation following severe traumatic brain injury. J Neurosurg.

[B90] Menon DK (2006). Brain ischemia after traumatic brain injury: lessons from ^15^O_2 _positron emission tomography. Curr Opin Crit Care.

[B91] Zazulia AR, Diringer MN, Videen TO, Adams RE, Yundt K, Aiyagari V, Grubb RL, Powers WJ (2001). Hypoperfusion without ischemia surrounding acute intracerebral hemorrhage. J Cereb Blood Flow Metab.

[B92] Reeves BC, Murphy GJ (2008). Increased mortality, morbidity, and cost associated with red blood cell transfusion after cardiac surgery. Curr Opin Cardiol.

[B93] Roach GW, Kanchuger M, Mangano CM, Neuman M, Nussmeier N, Wolman R, Aggarwal A, Marschall K, Graham SH, Ley C (1996). Adverse cerebral outcomes after coronary bypass surgery. Multicenter Study of Perioperative Ischemia Research Group and the Ischemia Research and Education Foundation Investigators. N Engl J Med.

[B94] McKhann GM, Grega MA, Borowicz LM, Baumgartner WA, Selnes OA (2006). Stroke and encephalopathy after cardiac surgery: an update. Stroke.

[B95] Newmann MF, Kirchner JL, Phillips-Bute B, Gaver V, Grocott H, Jones RH, Mark DB, Reves JG, Blumenthal JA (2001). Longitudinal assessment of neurocognitive function after coronary-artery bypass surgery. N Engl J Med.

[B96] Van Dijk D, Jansen EW, Hijman R, Nierich AP, Diephuis JC, Moons KG, Lahpor JR, Borst C, Keizer AM, Nathoe HM, Grobbee DE, DeJaegere PP, Kalkman CJ (2002). Cognitive outcome after off-pump and on-pump coronary artery bypass graft surgery: a randomized trial. JAMA.

[B97] Karkouti K, Wijeysundera DN, Beattie WS, Reducing Bleeding in Cardiac Surgery (RBC) Investigators (2008). Risk associated with preoperative anemia in cardiac surgery: a multicenter cohort study. Circulation.

[B98] Bell ML, Grunwald GK, Baltz JH, McDonald GO, Bell MR, Grover FL, Shroyer ALW (2008). Does preoperative hemoglobin independently predict short-term outcome after coronary artery bypass graft surgery?. Ann Thorac Surg.

[B99] Karkouti K, Wijeysundera DN, Yau TM, McCluskey SA, van Rensvurg A, Beattie WS (2008). The influence of baseline hemoglobin concentration on tolerance of anemia in cardiac surgery. Transfusion.

[B100] Chang YL, Tsai YF, Lin PJ, Chen MC, Liu CY (2008). Prevalence and risk factors for post-operative delirium in a cardiovascular intensive care unit. Am J Crit Care.

[B101] Kulier A, Levin J, Moser R, Rumpold-Seitlinger G, Tudor IC, Snyder-Ramos SA, Moehnle P, Mangano DT (2007). Impact of preoperative anemia on outcome in patients undergoing coronary artery bypass graft surgery. Circulation.

[B102] Matthew JP, Mackensen GB, Phillips-Bute B, Stafford-Smith M, Podgoreanu MV, Grocott HP, Hill SE, Smith PK, Blumenthal JA, Reves JG, Newman MF, for the Neruologic Outcome Research Group (NORG) of the Duke Heart Center (2007). Effects of extreme hemodilution during cardiac surgery on cognitive function in the elderly. Anesthesiology.

[B103] Cladellas M, Bruguera J, Cmoin J, Vila J, de Jaime E, Marti J, Gomez M (2006). Is pre-operative anemia a risk marker for in-hospial mortality and morbidity after valve replacement?. Eur Heart J.

[B104] Giltay EJ, Huijskes RV, Kho KH, Blansjaar BA, Rosseel PM (2006). Psychotic symptoms in patients undergoing coronary artery bypass grafting and heart valve operation. Eur J Cardiothorac Surg.

[B105] Karkouti K, Djaiani G, Borger MA, Beattie WS, Fedorko L, Wijeysundera D, Ivanov J, Karski J (2005). Low hematocrit during cardiopulmonary bypass is associated with increaed risk of perioperative stroke in cardiac surgery. Ann Thorac Surg.

[B106] Habib RH, Zacharias A, Schwann TA, Riordan CJ, Durham SJ, Shah A (2003). Adverse effects of low hematocrit during cardiopulmonary bypass in the adult: should current practice be changed. J Thorac Cardiovasc Surg.

[B107] DeFoe GR, Ross CS, Olmstead EM, Surgenor SD, Fillinger MP, Groom RC, Forest RJ, Pieroni JW, Warren CS, Bogosian ME, Krumholz CF, Clark C, Clough RA, Weldner PW, Lahey SJ, Leavitt BJ, Marrin CA, Charlesworth DC, Marshall P, O'Connor GT (2001). Lowest hematocrit on bypass and adverse outcomes associated with coronary artery bypass grafting. Ann Thorac Surg.

[B108] van Wermeskerken GK, Lardenoye JW, Hill SE, Grocott HP, Phillips-Bute B, Smith PK, Reves JG, Newman MF (2000). Intraoperative physiologic variables and outcome in cardiac surgery: Part II. Neurologic outcome. Ann Thorac Surg.

[B109] Klein AA, Nashef SA, Sharples L, Bottrill F, Dyer M, Armstrong J, Vuylsteke A (2008). A randomized controlled trial of cell salvage in routine cardiac surgery. Anesth & Analg.

[B110] Carless PA, Henry DA, Moxey AJ, O'Connell DL, Brown T, Fergusson DA (2006). Cell salvage for minimizing perioperative allogeneic blood transfusion. Cochrane Database Syst Rev.

[B111] Ferraris VA, Ferraris SP, Saha SP, Hessel EA, Haan CK, Royston BD, Bridges CR, Higgins RS, Despotis G, Brown JR, Spiess BD, Shore-Lesserson L, Stafford-Smith M, Mazer CD, Bennett-Guerrero E, Hill SE, Body S, Society of Thoracic Surgoeons Blood Conservation Guideline Task Force, Society of Cardiovascular Anesthesiologists Special Task Force on Blood Transfusion (2007). Perioperative blood transfusion and blood conservation in cardiac surgery: the Society of Thoracic Surgeons and The Society of Cardiovascular Anesthesiologists clinical practice guidelines. Ann Thorac Surg.

[B112] Taneja R, Fernandes P, Marwaha G, Cheng D, Bainbridge D (2008). Perioperative coagulation management and blood conservation in cardiac surgery: a Canadian Survey. J Cardiothorac Vasc Anesth.

[B113] Tinmouth AT, McIntyre LA, Fowlder RA (2008). Blood conservation strategies to reduce the need for red blood cell transfusion in critically ill patients. CMAJ.

[B114] Jonas RA, Wypij D, Roth SJ, Bellinger DC, Visconti KJ, du Plessis AJ, Goodkin H, Laussen PC, Farrell DM, Bartlett J, McGrath E, Rappaport LJ, Bacha EA, Forbess JM, del Nido PJ, Mayer JE, Newburger JW (2003). The influence of hemodilution on outcome after hypothermic cardiopulmonary bypass: results of a randomized trial in infants. J Thorac Cardiovasc Surg.

[B115] Newburger JW, Jonas RA, Soul J, Kussman BD, Bellinger DC, Laussen PC, Robertson R, Mayer JE, del Nido PJ, Bacha EA, Forbess JM, Pigula F, Roth SJ, Visconti KJ, du Plessis AJ, Farrell DM, McGrath E, Rappaport LA, Wypij D (2008). Randomized trial of hematocrit 25% versus 35% during hypothermic cardiopulmonary bypass in infant heart surgery. J Thorac Cardiovasc Surg.

[B116] Wypij D, Jonas RA, Bellinger DC, Del Nido PJ, Mayer JE, Bacha EA, Forbess JM, Pigula F, Laussen PC, Newburger JW (2008). The effect of hematocrit during hypothermic cardiopulmonary bypass in infant heart surgery: results from the combined Boston hematocrit trials. J Thorac Cardiovasc Surg.

[B117] Brevig J, McDonald J, Zelinka ES, Gallagher T, Jin R, Grunemeier GL (2009). Blood transfusion reduction in cardiac surgery: multidisciplinary approach at a community hospital. Ann Thorac Surg.

[B118] Ngaage DL, Cowen ME, Griffin S, Guvendik L, Cale AR (2008). Early neurological complications after coronary artery bypass grafting and valve surgery in octogenarians. Eur J Cardiothorac Surg.

[B119] Murphy GJ, Reeves BC, Rogers CA, Rizvi SI, Culliford L, Angelini GD (2007). Increased mortality, postoperative morbidity, and cost after red blood cell transfusion in patients having cardiac surgery. Circulation.

[B120] Whitson BA, Huddleston SJ, Savik K, Shumway SJ (2007). Bloodless cardiac surgery is associated with decreased morbidity and mortality. J Card Surg.

[B121] Norkiene I, Ringaitiene D, Misiureiene I, Samalavicuius R, Bubulis R, Baublys A, Uzdavinys G (2007). Incidence and precipitating factors of delirium after coronary artery bypass grafting. Scadn Cardiovasc J.

[B122] Koch CG, Li L, Duncan AI, Mihaljevic T, Cosgrove DM, Loop "FD, Starr NJ, Blackstone EH (2006). Morbidity and mortality risk associated with red blood cell and blood-component transfusion in isolated coronary artery bypass grafting. Crit Care Med.

[B123] Stamou SC, White T, Barnett S, Boyce SW, Corso PJ, Lefrak EA (2006). Comparisons of cardiac surgery outcomes in Jehovah's versus non-Jehovah's witnesses. Am J Cardiol.

[B124] Bucerius J, Gummert JF, Borger MA, Walther T, Doll N, Onnasch JF, Metz S, Falk V, Mohrt FW (2003). Stroke after cardiac surgery: a risk factor analysis of 16,184 consecutive adult patients. Ann Thorac Surg.

[B125] D'Ancona G, Saez de Ibarra JI, Baillot R, Mathieu P, Doyle D, Metras J, Desaulniers D, Dagenais F (2003). Determinants of stroke after coronary artery bypass grafting. Eur J Cardiothorac Surg.

[B126] Koch CG, Li L, Sessler DI, Figueroa P, Hoeltge GA, Mihaljevic T, Blackstone EH (2008). Duration of red-cell storage and complications after cardiac surgery. N Engl J Med.

[B127] Graham DI, Ford I, Adams JH, Doyle D, Teasdale GM, Lawrence AE, McLellan DR (1989). Ischemic brain damage is still common in fatal non-missile head injury. J Neurol Neurosurg Psychiatry.

[B128] Bouma GJ, Muizelaar JP, Stringer WA, Choi SC, Fatouros P, Young HF (1992). Ultra-early evaluation of reiongal cerebral blood flow in severely head-injured patients using xenon-enhanced computerized tomography. J Neurosurg.

[B129] Marion DW, Darby J, Yonas H (1991). Acute regional cerebral blood flow changes caused by severe head injuries. J Neurosurg.

[B130] Gopinath SP, Robertson CS, Contant CF, Hayes C, Feldman Z, Narayan RK, Grossman RG (1994). Jugular venous desaturation and outcome after head injury. J Neurol Neurosurg Psychiatry.

[B131] Van Santbrink H, Maas AI, Avezaat CJ (1996). Continuous monitoring of partial pressure of brain tissue oxygen in patients with severe head injury. Neurosurgery.

[B132] Valadka AB, Gopinath SP, Contant CF, Uzura M, Robertson CS (1998). Relationship of brain tissue PO2 to outcome after severe head injury. Crit Care Med.

[B133] Brink WA van den, van Santbrink H, Steyerberg EW, Avezaat CJ, Suazo JA, Hogesteeger C, Jansen WJ, Koos LM, Vermeulen J, Maas AI (2000). Brain oxygen tension in severe head injury. Neurosurgery.

[B134] Diringer MN, Videen TO, Yundt K, Zazulia AR, Aiyagari V, Dacey RG, Grubb RL, Powers WJ (2002). Regional cerebrovascular and metabolic effects of hyperventilation after severe traumatic brain injury. J Neurosurg.

[B135] Coles JP (2004). Regional ischemia after head injury. Curr Opin Crit Care.

[B136] Abate MG, Trivedi M, Fryer TD, Smielewski P, Chatfield DA, Williams GB, Aigbirhio F, Carpenter TA, Pickard JD, Menon DK, Coles JP (2008). Early derangements in oxygen and glucose metabolism following head injury: the ischemic penumbra and pathophysiological heterogeneity. Neurocrit Care.

[B137] Nortje J, Coles JP, Timofeev I, Fryer TD, Aigbirhio FI, Smielewsi P, Outtrim JG, Chatfield DA, Pichard JD, Hutchinson PJ, Gupta AK, Menon DK (2008). Effect of hyperoxia on regional oxygenation and metabolism after severe traumatic brain injury: preliminary findings. Crit Care Med.

[B138] Hare GM, Mazer CD, Hutchison JS, McLaren AT, Liu E, Rassouli A, Ai J, Shaye RE, Lockwood JA, Hawkins CE, Sikich N, To K, Baker AJ (2007). Severe hemodilutional anemia increases cerebral tissue injury following acute neurotrauma. J Appl Physiol.

[B139] Smith MJ, Stiefel MF, Magge S, Frangos S, Bloom S, Gracias V, Le Roux PD (2005). Packed red blood cell transfusion increases local cerebral oxygenation. Crit Care Med.

[B140] Leal-Noval SR, Rincon-Ferrari MD, Marin-Niebla A, Cayuela A, Arellano-Orden V, Marin-Caballos A, Amaya-Villar R, Ferrandiz-Millon C, Murillo-Cabeza F (2006). Transfusion of erythrocyte concentrates produces a variable increment on cerebral oxygenation in patients with severe traumatic brain injury: a preliminary study. Intensive Care Med.

[B141] Leal-Noval SR, Munoz-Gomez M, Aerllano-Orden V, Marin-Caballos A, Amaya-Villar R, Marin A, Puppo-Moreno A, Ferrandiz-Millon C, Flores-Cordero JM, Murillo-Cabezas F (2008). Impact of age of transfused blood on cerebral oxygenation in male patients with severe traumatic brain injury. Crit Care Med.

[B142] Zygun D, Nortje J, Hutchinson PJ, Timofeev I, Menon DK, Gupta AK (2009). Effect of red blood cell transfusion on cerebral oxygenation and metaoblism following severe trauamtic brain injury. Crit Care Med.

[B143] Carlson AP, Schermer CR, Lu SW (2006). Retrospective evaluation of anemia and transfusion in traumatic brain injury. J Trauma.

[B144] Steyerberg EW, Mushkuidani N, Perel P, Butcher I, Lu J, McHugh GS, Murray GD, Marmarou A, Robets I, Habbema JDF, Maas AIR (2008). Predicting outcome after traumatic brain injury: development and validation of international validation of prognostic scores based on admission characteristics. PLoS Med.

[B145] Duane TM, Mayglothling J, Grandhi R, Warrier N, Aboutanos MB, Wolfe LG, Malhotra AK, Ivatury RR (2008). The effect of anemia and blood transfusions on mortality in closed head injury patients. J Surg Res.

[B146] Salim A, Hadjizacharia P, DuBose J, Brown C, Inaba K, Chan L, Margulies DR (2008). Role of anemia in traumatic brain injury. J Am Coll Surg.

[B147] George ME, Skarda DE, Watts CR, Pham HD, Beilman GJ (2008). Aggressive red blood cell transfusion: no association with improved outcomes for victims of isolated traumatic brain injury. NeuroCrit Care.

[B148] Van Beek JGM, Mushkudiani NA, Steyerberg EW, Butcher I, McHugh GS, Lu J, Marmarou A, Murrary GD, Maas AIR (2007). Prognostic value of admission laboratory parameters in traumatic brain injury: results from the IMPACT Study. J Neurotrauma.

[B149] Schirmer-Makalsen K, Vik A, Gisvold SE, Skandsen T, Hynne H, Klepstad P (2007). Severe head injury: control of physiological variables, organ failure and complications in the intensive care unit. Acta Anaesthesiol Scand.

[B150] McIntyre LA, Fergusson DA, Hutchison JS, Pagliarello G, Marshall JC, Yetisir E, Hare GM, Hébert PC (2006). Effect of a liberal versus restrictive transfusion strategy on mortality in patients with moderate to severe head injury. NeuroCrit Care.

[B151] Robertson CS, Gopinath SP, Goodman JC, Contant CF, Valadka AB, Narayan RK (1995). SjvO2 monitoring in head-injured patients. J Neurotrauma.

[B152] Bendel P, Koivisto T, Kononen M, Hanninen T, Huarskainen H, Saari T, Vapalahti M, Hernesniemi J, Vanninen R (2008). MR imaging of the brain 1 year after aneurysmal subarachnoid hemorrhage: randomized study comparing surgical with endovascular treatment. Radiology.

[B153] Shimoda M, Takeuchi M, Tominaga J, Oda S, Kumasaka A, Tsugane R (2001). Asymptomatic versus symptomatic infarcts from vasospasm in patients with subarachnoid hemorrhage: serial magnetic resonance imaging. Neurosurgery.

[B154] Kramer AH, Zygun DA, Bleck TP, Dumont AS, Kassell NF, Nathan B (2009). Relationship between hemoglobin concentrations and outcomes across subgroups of patients with aneurysmal subarachnoid hemorrhage. NeuroCrit Care.

[B155] Naidech AM, Drescher J, Ault ML, Shaibani A, Batjer HH, Alberts MJ (2006). Higher hemoglobin is associated with less cerebral infarction, poor outcome, and death after subarachnoid hemorrhage. Neurosurgery.

[B156] Naidech AM, Jovanovic B, Wartenberg KE, Parra A, Ostapkovich N, Connolly ES, Mayer SA, Commichau C (2007). Higher hemoglobin is associated with improved outcome after subarachnoid hemorrhage. Crit Care Med.

[B157] Tseng MY, Hutchinson PJ, Kirkpatrick PJ (2008). Effects of fluid therapy following aneurismal subarachnoid hemorrhage: a prospective clinical study. Br J Neurosurg.

[B158] Wartenberg KE, Schmidt JM, Claassen J, Temes RE, Frontera JA, Ostapkovich N, Parra A, Connolly ES, Mayer SA (2006). Impact of medical complications on outcome after subarachnoid hemorrhage. Crit Care Med.

[B159] DeGeorgia M, Deogaonkar A, Ondrejka J, Katzan I, Sabharwal V, Woo HH, Rasmussen P, Chow M, Mayberg M Blood transfusionfollowing subarachnoid hemorrhage worsens outcome. http://www.abstractonline.com/arch/RecordView.aspx?LookupKey=12345&recordID=11885.

[B160] Smith MJ, Le Roux PD, Elliott JP, Winn HR (2004). Blood transfusion and increased risk for vasospasm and poor outcome after subarachnoid hemorrhage. J Neurosurg.

[B161] Sen J, Belli A, Albon H, Morgan L, Petzold A, Kitchen N (2003). Triple-H therapy in the management of aneurysmal subarachnoid hemorrhage. Lancet Neurol.

[B162] Ekelund A, Reinstrup P, Ryding E, Andersson AM, Molund T, Kristiansson KA, Romner B, Brandt L, Saveland H (2002). Effects of iso- and hypervolemic hemodilution on regional cerebral blood flow and oxygen delivery for patients with vasospasm after aneurysmal subarachnoid hemorrhage. Acta Neurochir.

[B163] Muench E, Horn P, Bauhuf C, Roth H, Philipps M, Hermann P, Quintel M, Schmiedek P, Vajkoczy P (2007). Effects of hypervolemia and hypertension on regional cerebral blood flow, intracranial pressure, and brain tissue oxygenation after subarachnoid hemorrhage. Crit Care Med.

[B164] Dhar R, Zazulia A, Videen T, Diringer M (2008). Red blood cell transfusion increases cerebral oxygen delivery after subarachnoid hemorrhage. NeuroCrit Care.

[B165] Oddo M, Milby A, Chen I, Frangos S, Maloney-Wilensky E, Stiefel M, Kofke WA, Levine JM, Le Roux PD (2009). Hemoglobin level and cerebral metabolism in patients with aneurysmal subarachnoid hemorrhage: a microdialysis study. Stroke.

[B166] Grotta J, Ackerman R, Correia J, Fallick G, Chang J (1982). Whole blood viscosity parameters and cerebral blood flow. Stroke.

[B167] Tohgi H, Yamanouchi H, Murakami M, Kameyama M (1978). Importance of the hematocrit as a risk factor in cerebral infarction. Stroke.

[B168] Roh JK, Kang DW, Lee SH, Yoon BW, Chang KH (2000). Significance of acute multiple brain infarction on diffusion-weighted imaging. Stroke.

[B169] Arauz A, Murillo L, Cantu C, Barinagarrementeria F, Higuera J (2003). Prospective study of single and multiple lacunar infarcts using magnetic resonance imaging: risk factors, recurrence, and outcome in 175 consecutive cases. Stroke.

[B170] Longo-Mbenza B, Lelo Tshinkwela M, Mbuilu Pukuta J (2008). Rates and predictors of stroke-associated case fatality in black Central African patients. Cardiovasc J Afr.

[B171] Kannel WB, Gordon T, Wolf PA, McNamara P (1972). Hemoglobin and the risk of cerebral infarction: the Framingham study. Stroke.

[B172] Gagnon DR, Zhang TJ, Brand FN, Kannel WB (1994). Hematocrit and the risk of cardiovascular disease – the Framingham study: a 34 year follow-up. Am Heart J.

[B173] Wannamethee G, Perry IJ, Shaper AG (1994). Hematocrit, hypertension and risk of stroke. J Intern Med.

[B174] Sacco S, Marini C, Olivieri L, Pistoia F, Carolei A (2007). Contribution of hematocrit to early mortality after ischemic stroke. Eur Neurol.

[B175] Diamond PT, Gale SD, Evans BA (2003). Relationship of initial hematocrit level to discharge destination and resource utilization after ischemic stroke: a pilot study. Arch Phys Med Rehabil.

[B176] Harrison MJ, Pollock S, Kendall BE, Marshall J (1981). Effect of hematocrit on carotid stenosis and cerebral infarction. Lancet.

[B177] Lowe GDO, Jaap AJ, Forbes CD (1983). Relation of atrial fibrillation and high hematocrit to mortality in acute stroke. Lancet.

[B178] Allport LE, Parsons MW, Butcher KS, MacGregor L, Desmond PM, Tress BM, Davis SM (2005). Elevated hematocrit is associated with reduced reperfusion and tissue survival in acute stroke. Neurology.

[B179] Huang WY, Chen IC, Meng L, Weng WC, Peng TI (2009). The influence of anemia on clinical presentation and outcome of patients with first-ever atherosclerosis-related ischemic stroke. J Clin Neurosci.

[B180] Huang WY, Weng WC, Chien YY, Wu CL, Peng TI, Chen KH (2008). Predictive factors of outcome and stroke recurrence in patients with unilateral atherosclerosis-related internal carotid artery occlusion. Neurol India.

[B181] Nybo M, Kristensen SR, Mickley H, Jensen JK (2007). The influence of anemia on stroke prognosis and its relation to N-terminal pro-brain natriuretic peptide. Eur J Neurol.

[B182] Bhatia RS, Garg RK, Gaur SPS, Kar AM, Shukla R, Agarwal A, Verma R (2004). Predictive value of routine hematological and biochemical parameters on 30-day fatality in acute stroke. Neurol India.

[B183] Wade JP, Taylor DW, Barnett HJ, Hachinksi VC (1987). Hemoglobin concentration and prognosis in symptomatic obstructive cerebrovascular disease. Stroke.

[B184] LaRue L, Alter M, Lai SM, Friday G, Sobel E, Levitt L, McCoy R, Isack T (1987). Acute stroke, hematocrit, and blood pressure. Stroke.

[B185] Kusonoki M, Kimura K, Nakamura M, Isaka Y, Yoneda S, Abe H (1981). Effects of hematocrit variations on cerebral blood flow and oxygen transport in ischemic cerebrovascular disease. J Cereb Blood Flow Metab.

[B186] Xiong L, Lei C, Wang Q, Li W (2008). Acute normovolemic hemodilution with a novel hydroxyethylstarch (130/0.4) reduces focal cerebral ischemic injury in rats. Eur J Anaesthesiol.

[B187] Lee SH, Heros RC, Mullan JC, Korosue K (1994). Optimum degree of hemodilution for brain protection in a canine model of focal cerebral ischemia. J Neurosurg.

[B188] Reasoner DK, Ryu KH, Hindman BJ, Cutkomb J, Smith T (1996). Marked hemodilution increases neurologic injury after focal cerebral ischemia in rabbits. Anesth Analg.

[B189] Kim JS, Kang SY (2000). Bleeding and subsequent anemia: a precipitant for cerebral infarction. Eur Neurol.

[B190] Bosel J, Ruscher K, Ploner CJ, Valdueza JM (2005). Delayed neurological deterioration in a stroke patients with postoperative acute anemia. Eur Neurol.

[B191] Shahar A, Sadeh M (1991). Severe anemia associated with transient neurological deficits. Stroke.

[B192] Asplund K (2002). Hemodilution for acute ischemic stroke. Cochrane Database Syst Rev.

[B193] Italian Acute Stroke Study Group (1998). Hemodilution in acute stroke: results from the Italian Hemodilution Trial. Lancet.

[B194] Scandinavian Stroke Study Group (1987). Multicenter trial of hemodilution in acute ischemic stroke: I. Results in the toal patient population. Stroke.

[B195] Goslinga H, Eijzenbach V, Heuvelmans JH, Laan de Vries E van der, Melis VM, Schmid-Schoenbein H, Bezemer PD (1992). Custom-tailored hemodilution with albumin and crystalloids in acute ischemic stroke. Stroke.

[B196] Aichner FT, Fazekas F, Brainin M, Polz W, Mamoli B, Zeiler K (1998). Hypervolemic hemodilution in acute ischemic stroke: the Multicenter Austrian Hemodilution Stroke Trial (MAHST). Stroke.

[B197] Belayev L, Busto R, Zhao W, Clemens JA, Ginsberg MD (1997). Effect of delayed albumin hemodilution on infarction volume and brain edema after transient middle cerebral artery occlusion in rats. J Neurosurg.

[B198] Palesch YY, Hill MD, Ryckborst KJ, Tamariz D, Ginsberg MD (2006). The ALIAS Pilot Trial: a dose-escalation and safety study of albumin therapy for acute ischemic stroke – II: neurologic outcome and efficacy analysis. Stroke.

[B199] Ginsberg MD, Hill MD, Palesch YY, Ryckborst KJ, Tamariz D (2006). The ALIAS Pilot Trial: a dose-escalation and safety study of albumin therapy for acute ischemic stroke – I: physiological responses and safety results. Stroke.

[B200] Shin DH, Moon GJ, Bang OY (2007). Albumin therapy in acute stroke patients. J Neurol.

[B201] Mayer SA, Rincon F (2005). Management of intracerebral hemorrhage. Lancet Neurol.

[B202] Mendelow AD (1993). Mechanisms of ischemic damage with intracerebral hemorrhage. Stroke.

[B203] Siddique MS, Fernandes HM, Wooldridge TD, Fenwick JD, Slomka P, Mendelow AD (2002). Reversible ischemia around intracerebral hemorrhage: a single-photon emission computerized tomography study. J Neurosurg.

[B204] Schellinger PD, Fiebach JB, Hoffmann K, Becker K, Orakcioglu B, Kollmar R, Juttler E, Schramm P, Schwab S, Sartor K, Hacke W (2003). Stroke MRI in intracerebral hemorrhage: is there a perihemorrhagic penumbra?. Stroke.

[B205] Rosand J, Eskey C, Chang Y, Gonzalez RG, Greenberg SM, Koroshetz WJ (2002). Dynamic single-section CT demonstrates reduced cerebral blood flow in acute intracerebral hemorrhage. Cerebrovasc Dis.

[B206] Herweh C, Juttler E, Schellinger PD, Klotz E, Jenetzky E, Orakcioglu B, Sartor K, Schramm P (2007). Evidence against a perihemorrhagic penumbra provided by perfusion computed tomography. Stroke.

[B207] Nilsson OG, Polito A, Saveland H, Ungerstedt U, Norstrom CH (2006). Are the primary supratentorial intracerebral hemorrhages surrounded by a biochemical penumbra? A microdialysis study. Neurosurgery.

[B208] Aronowski J, Strong R, Grotta JC (1996). Combined neuroprotection and reperfusion therapy for stroke. Effect of lubeluzole and diaspirin cross-linked hemoglobin in experimental focal ischemia. Stroke.

[B209] Bowes MP, Burhop KE, Zivin JA (1994). Diaspirin cross-linked hemoglobin improves neurological outcome following reversible but not irreversible CNS ischemia in rabbits. Stroke.

[B210] Cole DJ, Drummon JC, Patel PM, Reynolds LR (1997). Hypervolemic-hemodilution during cerebral ischemia in rat: effects of diaspirin cross-linked hemoglobin (DCLHb) on neurological outcome and infarct volume. J Neurosurg Anesthesiol.

[B211] Mito T, Nemoto M, Kwansa H, Samepi K, Habeeb M, Murphy SJ, Bucci E, Koehler RC (2009). Decreased damage from transient focal cerebral ischemia by transfusion of zero-link hemoglobin polymers in mouse. Stroke.

[B212] Nemoto M, Mito T, Brinigar WS, Fronticelli C, Koehler RC (2006). Salvage of focal cerebral ischemic damage by transfusion of high O_2_-affinity recombinant hemoglobin polymers in mouse. J Appl Physiol.

[B213] Piper IR, Garrioch MA, Souter MJ, Andrews PJ, Thomson D (1998). Effects of diaspirin cross-linked hemoglobin on post-traumatic cerebral persuion pressure and blood flow in a rodent model of diffuse brain injury. Br J Anaesth.

[B214] Gibson JB, Maxwell RA, Schwiezter JB, Fabian TC, Proctor KG (2002). Resuscitation from severe hemorrhagic shock after tramatic brain injury using saline, shed blood, or a blood substitute. Shock.

[B215] Chappell JE, Shackford SR, McBride WJ (1997). Effect of hemodilution with diaspirin cross-linked hemoglobin on intracranial pressure, cerebral perfusion pressure, and fluid reuirements after head injury and shock. J Neurosurg.

[B216] Dudkiewicz M, Harpaul TA, Proctor KG (2008). Hemoglobin-based oxygen carrying compound-201 as salvage therapy for severe neuro-and polytrauma (Injury Severity Score = 27–41). Crit Care Med.

[B217] Stern S, Rice J, Philbin N, McGwin G, Arnaud F, Johnson T, Flournoy WS, Ahlers S, Pearce LB, McCarron R, Freilich D (2009). Resuscitation with the hemoglobin-based oxygen carrier, HOBC-201, in a swine model of severe uncontrolled hemorrhage and traumatic brain injury. Shock.

[B218] Rosenthal G, Morabito D, Cohen M, Roeytenberg A, Derugin N, Panter SS, Knodson MM, Maley G (2008). Use of hemoglobin-based oxygen-carrying solution-201 to improve resuscitation parameters and prevent secondary brain injury in a swine model of traumatic brain injury and hemorrhage; laboratory investigation. J Neurosurg.

[B219] Kerby JD, Sainz JG, Zhang F, Hutchings A, Sprague S, Farrokhi FR, Son M (2007). Resuscitation from hemorrhagic shock with HOBC-201 in the setting of traumatic brain injury. Shock.

[B220] Cole DJ, Reynolds LW, Nary JC, Drummond JC, Patel PM, Jacobsern WK (1999). Subarachnoid hemorrhage in rats: effect of singular or sustained hemodilution with alpha-alpha diaspirin crosslinked hemoglobin on cerebral hypoperfusion. Crit Care Med.

[B221] Cole DJ, Nary JC, Reynolds LW, Patel PM, Drummond JC (1997). Experimental subarachnoid hemorrhage in rats: effect of intravenous alpha-alpha diaspitin crosslinked hemoglobin on hypoperfusion and neuronal death. Anesthesiology.

[B222] Rother RP, Bell L, Hillmen P, Gladwin MT (2005). The clinical sequelae of intravascular hemolysis and extracellular plasma hemoglobin: a novel mechanism of human disease. JAMA.

[B223] Natanson C, Kern SJ, Lurie P, Banks SM, Wolfe SM (2008). Cell-free hemoglobin-based blood substitutes and risk of myocardial infarction and death. JAMA.

[B224] Saxena R, Wihnhoud AD, Carton H, Hacke W, Kaste M, Przybelski RJ, Stern KN, Koudstaal PJ (1999). Controlled safety study of a hemoglobin-based oxygen carrier, DCLHb, in acute ischemic stroke. Stroke.

[B225] Kerner T, Ahlers O, Veit S, Riou B, Saunders M, Pison U, for the European DCLHb Trauma Study Group (2003). DCL-Hb for trauma patients with severe hemorrhagic shock: the European "On-Scene" Multicenter Study. Intensive Care Med.

[B226] Sloan EP, Koenigsberg M, Gens D, Cipolle M, Runge J, Mallory MN, Rodman G, for the DCLHb Traumatic Hemorrhagic Shock Study Group (1999). Diaspirin cross-linked hemoglobin (DCLHb) in the treatment of severe traumatic hemorrhagic shock: a randomized controlled efficacy trial. JAMA.

[B227] Gould SA, Moore EE, Hoyt DB, Burch JM, Haenel JB, Garcia J, DeWoskin R, Moss GS (1998). The first randomized trial of human polymerized hemoglobin as a blood substitute in acute trauma and emergent surgery. J Am Coll Surg.

[B228] Przybelski RJ, Daily EK, Micheels J, Sloan E, Mols P, Corne L, Koenigerg MD, Bickell WH, Thompson DR, Harviel JD, Cohn SM (1999). A safety assessment of diaspirin cross-linked hemoglobin (DCLHb) in the treatment of hemorrhagic, hypovolemic shock. Prehosp Disaster Med.

[B229] Moore EE, Moore FA, Fabian TC, Bernard AC, Fulda GJ, Hoyt DB, Duane TM, Weireter LJ, Gomez GA, Ciplle MD, Rodman GH, Malangoni MA, Hides GA, Omert LA, Gould SA (2009). Human polymerized hemoglobin for the treatment of hemorrhagic shock when blood is unavailable: the USA multicenter trial. J Am Coll Surg.

[B230] Lamy ML, Daily EK, Brichant JF, Larbuisson RP, Demeyere RH, Vandermeersch EA, Lehot JJ, Parsloe MR, Berridge JC, Sinclair CJ, Baron JF, Przybelski RJ, for the DCLHb Cardiac Surgery Trial Collaborative Group (2000). Randomized trial of diaspirin cross-linked hemoglobin solution as an alternative to blood transfusion after cardiac surgery. Anesthesiology.

[B231] Hill SE, Gottshcalk LI, Grichnik K (2002). Safety and preliminary efficacy of hemoglobin raffimer for patients undergoing coronary artery bypass surgery. J Cardiothorac Vasc Anesth.

[B232] Lindsay P, Bayley M, Hellings C, Hill M, Woddbury E, Phillips S (2008). Canadian best practice recommendations for stroke care (updated 2008). CMAJ.

[B233] Adams HP, del Zoppo G, Alberts MJ, Bhatt DL, Brass L, Furlan A, Grubb RL, Higashida RT, Jauch EC, Kidwell C, Lyden PD, Morgenstern LB, Qureshi AI, Rosenwasser RH, Scott PA, Wijdicks EF, American Heart Association; American Stroke Association Stroke Council; Clinical Cardiology Council; Cardiovascular Radiology and Intervention Council; Atherosclerotic Peripheral Vascular Disease and Quality of Care Outcomes in Research Interdisciplinary Working Groups (2007). Guidelines for the early management of adults with ischemic stroke: a guideline from the American Heart Association/American Stroke Association Stroke Council, Clinical Cardiology Council, Cardiovascular Radiology and Intervention Council, and the Atherosclerotic Peripheral Vascular Disease and Quality of Care Outcomes in Research Interdisciplinary Working Groups: the American Academy of Neurology affirms the value of this guideline as an educational tool for neurologists. Stroke.

[B234] Brain Trauma Foundation; American Association of Neurological Surgeons; Congress of Neurological Surgeons (2007). Guidelines for the management of severe traumatic brain injury. J Neurotrauma.

[B235] Bederson JB, Connolly ES, Batjer HH, Dacey RG, Dion JE, Diringer MN, Duldner JE, Harbaugh RE, Patel AB, Rosenwasser RH (2009). Guidelines for the management of aneurysmal subarachnoid hemorrhage. Stroke.

[B236] Broderick J, Connolly S, Feldmann E, Hanley D, Kase C, Krieger D, Mayberg M, Morgenstern L, Ogilvy CS, Vespa P, Zuccarello M, American Stroke Association Stroke Council; High Blood Pressure Research Council; Quality of Care and Outcomes in Research Interdisciplinary Working Group (2007). Guidelines for the management of spontaneous intracerebral hemorrhage in adults: 2007 update: a guideline from the American Heart Association/American Stroke Association Stroke Council, High Blood pressure Research Council, and the Quality of Care and Outcomes in Research Interdisciplinary Working Group. Stroke.

[B237] Chang JJ, Youn TS, Benson D, Mattick H, Andrade BA, Harper MS, Moore CB, Madden CJ, Diaz-Arrastia RR (2009). Physiologic and functional outcome correlates of brain tissue hypoxia in traumatic brain injury. Crit Care Med.

[B238] Naidech AM, Bendok BR, Ault ML, Bleck TP (2008). Monitoring with the Somanetics INVOS 5100C after aneurysmal subarachnoid hemorrhage. Neurocrit Care.

[B239] Sahuquillo J, Poca MA, Garnacho A, Robles A, Coello F, Godet C, Triginer C, Rubio E (1993). Early ischemia after severe head injury. Preliminary results in patients with diffuse brain injuries. Acta Neurochir.

[B240] Cruz J, Jaggi JL, Hoffstad OJ (1993). Cerebral blood flow and oxygen consumption in acute brain injury with acute anemia: an alternative for the cerebral metabolic rate of oxygen consumption?. Crit Care Med.

